# A prenucleation strategy for ambient fabrication of perovskite solar cells with high device performance uniformity

**DOI:** 10.1038/s41467-020-14715-0

**Published:** 2020-02-21

**Authors:** Kai Zhang, Zheng Wang, Gaopeng Wang, Jian Wang, Yu Li, Wei Qian, Shizhao Zheng, Shuang Xiao, Shihe Yang

**Affiliations:** Guangdong Provincial Key Lab of Nano-Micro Materials Research, School of Chemical Biology and Biotechnology, Shenzhen Graduate School, Peking University, Shenzhen, China

**Keywords:** Devices for energy harvesting, Solar energy, Solar cells

## Abstract

Humidity is known to be inimical to the halide perovskites and thus typically avoided during fabrication. The poor fundamental understanding of chemical interactions between water and the precursors hampers the further development of perovskite fabrication in ambient atmosphere. Here, we disclose a key finding that the ambient water could promote the formation of lead complexes, which when uncontrolled would make their way into large intermediate fibrillar crystallites and thus discontinuous perovskite films unfavorable for photovoltaics among others. To counter this effect, a prenucleation strategy is proposed, which embodies the controlled burst of profuse intermediate nuclei. Consequently, we are able to obtain a compact and uniform perovskite layer, which affords high efficiency perovskite solar cells. More excitingly, the solar cells show high performance uniformity, demonstrating the distinctive advantages of our prenucleation strategy. This work sheds light on developing reliable and cost-effective fabrication methods for industrial production of perovskite solar cells.

## Abstract (web summary)

Ambient processing of perovskite solar cells is desired but the resulting cell performance is poor due to the negative effects of moisture on film fabrication. Here Zhang et al. propose a prenucleation strategy to overcome the moisture effect, achieving good film quality and high and uniform cell performance.

## Introduction

The organometal halide perovskites have emerged as one of the most competitive light absorbers for photovoltaic (PV) applications^1–6^. The certified power conversion efficiency (PCE) of perovskite solar cells (PVSCs) has now reached 25.2% for single junction cells and 29.1% for two terminal tandem cells, already outperforming the multi-crystalline Si solar cells (23.3%), CdTe thin film solar cells (22.1%), etc.^7^. More significantly, this outstanding efficiency comes at an extremely low cost since the perovskite solar cells can be obtained by a solution-based fabrication process, triggering a global competition to translate the brilliant perovskite materials to innovative technologies^8–11^. However, researchers quickly found that the performance of PVSCs is quite sensitive to humidity, oxygen and solvent vapors, severely restricting the fabrication condition of such solar cells^12–14^. Normally, the oxygen and H_2_O should be below the ppm level to avoid their perceptibly negative effects on the perovskite based devices. Clearly, the cost to maintain this condition is large and thus obstructive to the commercialization of PVSCs.

To tackle this issue, it is highly desirable to develop viable approaches to fabricating PVSCs in the ambient atmosphere condition. Conceivably, the biggest problem of ambient fabrication methods would be the efficiency loss due to interactions of the ambient water and oxygen with the perovskite precursors and substrate. A straightforward solution could be to heat the substrate or precursor to avoid the influence of humidity. Indeed, a thermal radiation protocol was used to heat up the surrounding air to create a low relative humidity (RH) field. Combined with 80 °C substrates, a dense perovskite layer could be fabricated in ambient air^13,15^. Nevertheless, the fast solvent evaporation leaves little time for the film formation, which would require extremely fine control over the fabrication conditions. For the solvent dripping method, humid ambient fabrication could also be achieved by adjusting the anti-solvent composition^12^. For example, ethyl acetate was found to be a better anti-solvent than toluene, diethyl ether and CB, as it is able to sequester airborne moisture and protect the perovskite precursor’s intermediate during the spin-coating process. However, due to the complex external environment, the temperature distribution and solvent evaporation rate could be easily influenced by such factors as airflow or even the operators. Against this backdrop, it would be desirable to develop a more controllable approach for fabricating perovskite devices.

An alternative way is to design chemical reaction routes, which could also eliminate the influence of humidity. One example along this direction include the use of a two-step method employing SCN^−^ for strong coordination to Pb^2+^ and hydrogen bond with CH_3_NH_3_^+^^16^. As another example, nanorods were converted to perovskite films in air^17^. Although these chemical reaction routes showed potential to realize the ambient fabrication of PVSCs, the inherent mechanisms are still unclear, and the variations of device performance to environmental conditions need to be improved. Thus, it remains a great challenge to rationally design chemical reactions or optimize fabrication strategies. Undoubtedly, fundamental understanding about the chemical interactions between perovskite precursors and ambient air should help to uncover the hidden variables for advancing the ambient fabrication approaches towards highly efficient PVSCs.

Herein, we present a prenucleation method for fabricating PVSCs in ambient humid atmosphere, which in effect can turn on its head the commonly nagging influence of the environmental species, such as water, oxygen and impurities. The method was developed based on the fundamental understanding of water promoted intermediate formation and conversion in the fabrication process of perovskite films. Here the prenucleation specifically refers to the controlled formation of a vast number of lead complexes during the wet sample spinning process. The characterization and calculation results allowed us to conclude that water facilitates the formation of the lead complexes, and even more, the uncontrolled growth of the large 1D intermediate crystallites, which could be only converted to discontinuous perovskite films unfavorable for photovoltaics. This scenario is significantly different from what has been reported previously, wherein only a limited amount of water was used as an additive to improve the performance of perovskite solar cells^4,8,12^. To overturn the harmful effect of excess water, we herein propose a prenucleation method, which embodies the introduction of a burst of the lead complexes to facilitate and prolong nucleation of the intermediate, affording plenty of suitable sized intermediate crystallites. In this way, the quasi-solid precursor film with such profuse intermediate crystallites could be readily converted to a high-quality solid perovskite thin film. This innovation has led to a conspicuous performance improvement for p-i-n PVSCs with the FTO/NiO_x_/MAPbI_3_/PMMA/PCBM/PPDIN6/Ag device structure, delivering an impressive PCE of 19.5%. More importantly, the PVSCs fabricated using this method but under different conditions delivered nearly the same performance, simply because the conversion process to perovskite proceeded via the controllably formed precursor film, which is nearly independent of the ambient environment. This high device performance uniformity resulting from the prenucleation strategy is a crucial property required for industrial production.

## Results

### Water promoted intermediates formation

**Fig. 1:**
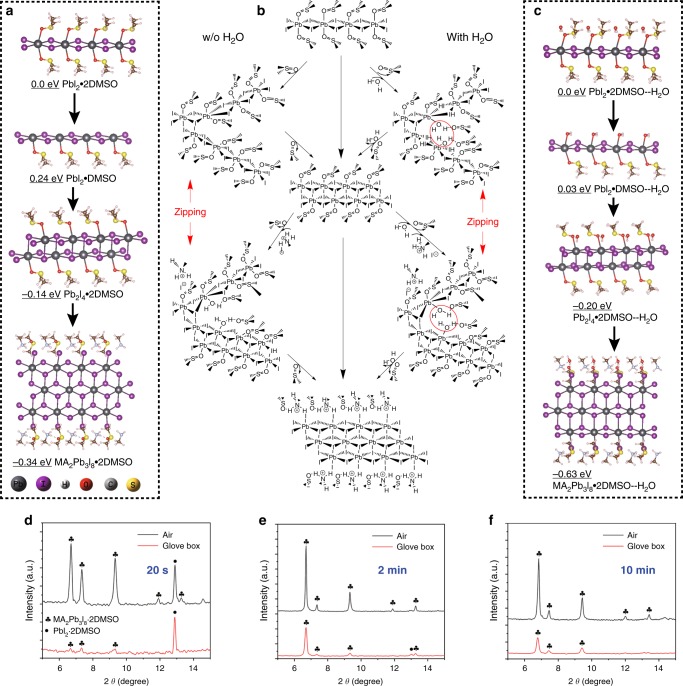
Water promoted formation of lead complexes—the prenucleation clusters. Calculated structures and electronic energies of lead complexes without H_2_O (**a**) or with H_2_O (**c**). **b** Schematic illustration of the plausible pathways to the formation of lead complexes, in much the same way as a zipping process. The lead complexes were formed in the absence (left column) and in the presence (right column) of water. The X-ray diffraction patterns (XRD) of Pb-complex films fabricated in ambient air and in glove box with the sample spinning time of **d** 20 s, **e** 2 min and **f** 10 min. The higher Pb-oligomers are easier to form in the presence of H_2_O than in the absence of H_2_O due to the H_2_O-enhanced stabilization and H_2_O-promoted removal of DMSO. Note that the Pb_2_I_4_·2DMSO oligomer was not observed in the sample spinning process due to the facile conversion to the MA_2_Pb_3_I_8_·2DMSO intermediate caused by the fast solvent evaporation. Nevertheless, the Pb_2_I_4_·2DMSO oligomer was observed by simply adding water without spinning the sample (see main text and XRD patterns in Supplementary Fig. 3).

The perovskite film synthesized using the conventional solvent dripping method in ambient air was rough and greyish black, inappropriate for fabricating high efficiency solar cells, in stark contrast to the mirror-like and shining black film synthesized in dry air glovebox (Supplementary Fig. 1). Obviously, the major difference between the ambient air and the dry air glove box was the different amount of water vapor depending on the humidity, indicating that water may play a key role in forming the film of poor quality. The effect of O_2_ could be ignored as will be shown below (Supplementary Fig. 1c). In other words, the chemical interactions between water and the components in perovskite precursors may influence the film formation. To understand these interactions, we performed comparative computational studies to analyze the perovskite film formation processes. Fig. 1a, b, c shows the different scenarios of complex formation in dry air glove box and in ambient air. When the MAI/PbI_2_ is dissolved in DMF/DMSO mixed solution, DMSO as a Lewis base will covalently bond to PbI_2_ to form a 1D complex (PbI_2_·2DMSO), which we term prenucleation cluster (Fig. 1b, left column)^18^. If no water is present in the solution, the complexes will eliminate DMSO and join together to form a larger complex (Pb_2_I_4_·2DMSO), that is, a larger prenucleation cluster, as the concentration increases. Further increase in concentration will lead to the nucleation, by combining with MAI, to form a key oligomeric intermediate (MA_2_Pb_3_I_8_·2DMSO) (Fig. 1b, left column)^10,18^ for the facile, perhaps nearly topotactical, conversion to MAPbI_3_ in a heat up process.

Now we turn to the situation in ambient air under otherwise the same conditions. The precursor (MAI + PbI_2_ in DMSO:DMF = 7:2) we used absorbed water vapor and formed a wet film^19^. According to our calculation, since the H_2_O molecule has strong hydrogen bond interaction with DMSO, it could break the O-Pb bond in the PbI_2_·2DMSO complex and form the water-bearing PbI_2_·DMSO--H_2_O complex (Fig. 1b, right column). As the concentration increases, the PbI_2_·DMSO--H_2_O could dissociate the H_2_O and connect another PbI_2_·DMSO--H_2_O to form the Pb_2_I_4_·2DMSO--H_2_O oligomer. Subsequently, the Pb_2_I_4_·2DMSO--H_2_O could dissociate the H_2_O and connect yet another PbI_2_·DMSO--H_2_O and MAI to form the MA_2_Pb_3_I_8_·2DMSO--H_2_O intermediate. Interestingly, the conversion from PbI_2_·2DMSO complex to Pb_2_I_4_·2DMSO oligomer and from Pb_2_I_4_·2DMSO oligomer to MA_2_Pb_3_I_8_·2DMSO intermediate is much like a zipping process in action (Fig. 1b, right column). Note that the DMSO and H_2_O molecules should be eliminated once the ‘zipper’ is closed. Because H_2_O is smaller than DMSO, it offers a smaller steric hindrance than DMSO does, making it easier and neater to close the ‘zipper’ (Fig. 1b, red circles). To further understand the difference of conversion from PbI_2_·2DMSO complex to MA_2_Pb_3_I_8_·2DMSO intermediate between inert environment and ambient air, the electronic energy of each intermediate state was calculated. In the anhydrous environment, the electronic energy of PbI_2_·DMSO complex is 0.24 eV higher than that of the PbI_2_·2DMSO complex (0.0 eV), indicating the relative difficulty of eliminating DMSO from PbI_2_·2DMSO (Fig. 1a). This would make it more difficult to form the two larger complexes. Thus, extra energy is needed to spit out DMSO from PbI_2_·2DMSO in the anhydrous environment, such as accelerating volatilization using spin coating or blending in anti-solvent^18^. However, in the ambient air, H_2_O·PbI_2_·DMSO complex would form, since it has a significantly lower electronic energy (0.03 eV). Clearly, H_2_O can play a promoting role in removing DMSO from PbI_2_·2DMSO (Fig. 1a, right column). Thus, the conversion from H_2_O·PbI_2_·2DMSO to H_2_O·PbI_2_·DMSO would proceed spontaneously in ambient air. This is also the case for conversion to the larger oligomers, meaning that H_2_O could promote the whole conversion process from the PbI_2_·2DMSO complex to the MA_2_Pb_3_I_8_·2DMSO intermediate. In summary, the simulation results suggest that the formation of the MA_2_Pb_3_I_8_·2DMSO intermediate is facilitated with the assistance of H_2_O.

To confirm the simulation results, we examined compositions of the intermediate complexes formed during the spin coating process. The X-ray diffraction patterns (XRD) showed that a large amount of the MA_2_Pb_3_I_8_·2DMSO intermediate and the PbI_2_·2DMSO complex formed after spinning for only 20 s in ambient air (Fig. 1d). However, only a very small quantity of the PbI_2_·2DMSO complex formed and the MA_2_Pb_3_I_8_·2DMSO intermediate could be hardly detected in dry air glove box under otherwise the same operating condition. After a 2 min spin coating process in ambient air, all the PbI_2_·2DMSO complexes were converted into the MA_2_Pb_3_I_8_·2DMSO intermediate in ambient air, whereas some PbI_2_·2DMSO complexes still remained unconverted during the same spin-coating process but in glove box (Fig. 1e). The characteristic XRD peak of MA_2_Pb_3_I_8_·2DMSO at 6.70° was chosen for comparison. The intermediate film fabricated in air (Air-INT) exhibits a relative peak intensity of 652 and full width of half maximum (FWHM) value of 0.147°, whereas for that fabricated in glovebox (GB-INT), the relative peak intensity is only 50 and the FWHM value is up to 0.367° (Fig. 1d). This result points to the lower crystallinity of GB-INT than that of Air-INT. When the spin coating time was prolonged to 2 min, the peak intensity of Air-INT and GB-INT increased to 4337 and 1905, respectively, while the corresponding FWHM value decreased to 0.089° and 0.147° (Fig. 1e). Thus, prolonging the spin coating time increases the crystallinity of both Air-INT and GB-INT, but the crystallinity of Air-INT is still higher than that of GB-INT. Even after prolonging the spin coating time to 10 min, the amount of the MA_2_Pb_3_I_8_·2DMSO intermediate formed in ambient air was still obviously larger than formed in glove box (Fig. 1f), further confirming the role of water in promoting the Pb intermediate growth. It is noteworthy that the solute concentration plays a critical role in the formation of the complexes and the intermediate. The Pb_2_I_4_·2DMSO could only be formed at low solute concentrations, whereas the MA_2_Pb_3_I_8_·2DMSO typically formed at high solute concentrations^18^. In the spin-coating process, the solute concentration increased as the solvent evaporated, so only the MA_2_Pb_3_I_8_·2DMSO intermediate could form but not the Pb_2_I_4_·2DMSO--H_2_O oligomer, as was observed.

To slow down the solvent evaporation, we added water into the precursor solution without spinning. Now we could indeed observe the conversion from PbI_2_·2DMSO to Pb_2_I_4_·2DMSO and then to MA_2_Pb_3_I_8_·2DMSO, in reasonable agreement with the simulation prediction (Supplementary Fig. 3a). In order to eliminate the influence of MAI and DMF, we only added water into a PbI_2_/DMSO solution, and in this way, the transition process from PbI_2_·2DMSO to Pb_2_I_4_·2DMSO could be observed more clearly (Supplementary Fig. 3b). To further illustrate this picture, we took FTIR spectra of PbI_2_ in DMSO solution as a function of time and of DMSO/H_2_O mixture to track the changes due to water (Supplementary Fig. 4). The result shows that water assisted in the formation of PbI_2_·2DMSO and the conversion from PbI_2_·2DMSO to Pb_2_I_4_·2DMSO. For the initial PbI_2_ in DMSO solution without water, the spectrum only showed DMSO and DMSO/PbI_2_ interaction (Supplementary Fig. 4a, black curve). After 5 min exposure in humid air, the solution absorbed water vapor, resulting in the spectral features of DMSO/H_2_O and DMSO/H_2_O/PbI_2_ interactions in place of the DMSO and the DMSO/PbI_2_ interaction (Supplementary Fig. 4a, red curve). As the exposure time was prolonged to 12 min, the DMSO/H_2_O and DMSO/H_2_O/PbI_2_ features became stronger, indicating a larger amount of water absorbed (Supplementary Fig. 4a, green curve). The results above firmly prove that the MA_2_Pb_3_I_8_·2DMSO intermediate is more easily formed in ambient air than in glove box due to the assistance of water.

**Fig. 2:**
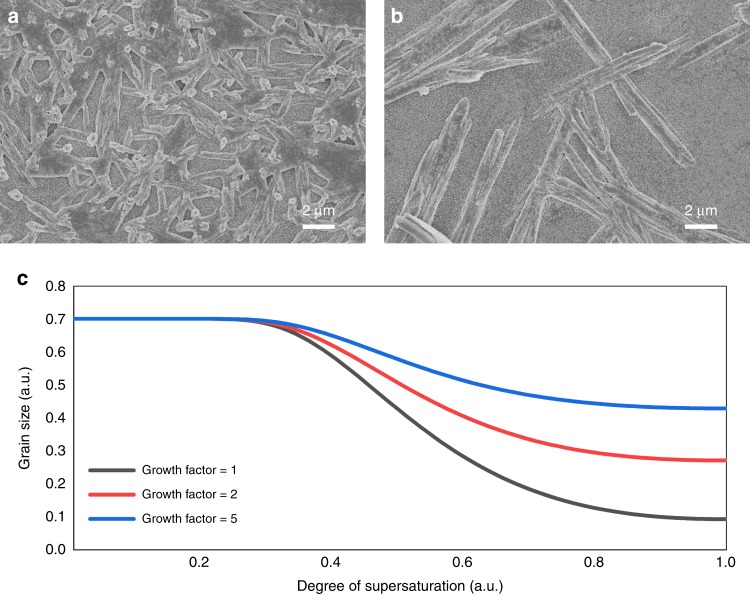
The intermediate formation in dry air and in ambient atmosphere. The scanning electron microscope (SEM) image of the MA_2_Pb_3_I_8_·2DMSO intermediate in **a** glove box and **b** ambient atmosphere without the antisolvent dripping step (spinning at 6000 r.p.m. for 2 min and annealing at 98 °C for 1 min). The rods in the sample fabricated in ambient atmosphere have a lower density but a larger size than that fabricated in glove box. **c** The simulated crystal size as a function of the supersaturation degree, with different growth factors. In general, the crystal size decreases as the growth factor decreases or as the supersaturation degree increases. A larger growth factor means that the growth rate increases more than the nucleation rate, when the crystallization condition is changing.

The morphology of Pb-complexes synthesized in dry air glove box and in ambient air was compared to further bolster the theory behind our prenucleation strategy. After spinning the sample at 6000 r.p.m. for 2 min and annealing at 98 °C for 1 min, we could obtain the MA_2_Pb_3_I_8_·2DMSO intermediate with a rod-like morphology^10,18^. The intermediates synthesized in glove box were rod-like and with the diameter around 0.6 μm and length around 4 μm (Fig. 2a). However, the intermediates synthesized in ambient air were much larger with the diameter around 1.5 μm and length over 12 μm (Fig. 2b). These intermediates are uniform in large area, indicating that the morphology in Fig. 2a and b is not a local effect (Supplementary Fig. 5). One can imagine that larger sized intermediate crystallites are more difficult to be converted to perovskite crystallites, because the conversion inevitably suffers a shape change from the 1D intermediate to the 3D perovskite due to the intrinsically different crystallization habits. Moreover, the number of nuclei formed in ambient air was far less than that in dry air glove box. These results also prove that the MA_2_Pb_3_I_8_·2DMSO intermediate is easier to grow to a large size in ambient air than in glove box due to the assistance of water.

To better understand the effect of water vapor on the film morphologies, it is instructive to apply the Volmer-Weber theory to the abovementioned two cases^20^. In the Volmer-Weber growth mode, the nuclei form separately and grow into ‘islands’ on a substrate due to the strong adatom-adatom interaction^21,22^. Then those islands continue to grow, connect to each other and finally form a continuous film. The nucleation and growth processes are highly dependent on the nucleation and growth rates of the intermediate, which is so critical that could allow a topotactical conversion to high-quality perovskite films. According to the Burton-Cabrera-Frank (BCF) theory, the number of crystallites per unit area (*N*) can be expressed as^23–25^:

1 $$N = 1.1\left( {\frac{{V_1}}{{V_2}}} \right)^{1/2}$$

here *V*_1_ is the nucleation rate and has an exponential relation to the supersaturation *σ*, i.e., *V*_1_ ∝ exp(−1/*σ*^2^), *V*_2_ is the crystal growth rate, which is related to the supersaturation as *V*_2_ ∝ *σ*^2^ and *V*_2_ ∝ *σ* at a low and high degree of supersaturation, respectively. Since *N* is inversely proportional to the crystallite size, we can simulate the crystallite size as a function of supersaturation degree, and the result is shown in Fig. 2c. In light of the discussion above, water can promote the nucleation and growth of the MA_2_Pb_3_I_8_·2DMSO intermediate by reducing the steric hindrance and creating a smooth downhill zipping kinetics. In other words, water increases the nucleation and growth rates of the MA_2_Pb_3_I_8_·2DMSO intermediate. For clarity of discussion, here we define a parameter, ‘growth factor (*F*_g_)’, to describe the relative increment of growth rate compared to the nucleation rate due to the presence of water. Thus, the modified equation for the number of crystallites per unit area now becomes:

2 $$N = 1.1\left( {\frac{{V_1}}{{F_{\mathrm{g}} \ast V_2}}} \right)^{1/2}$$

Obviously, if the growth rate increases faster than the nucleation rate, the growth factor will be larger than 1, and the faster it increases, the lager the growth factor. The simulated result in Fig. 2c clearly shows that the crystallite size increases with the growth factor increases, meaning that water has a stronger promotion effect on the growth rate than the nucleation rate. Plausibly, in ambient atmosphere, water could be absorbed into the wet film, and this could increase the MA_2_Pb_3_I_8_·2DMSO intermediate growth rate more quickly than the nucleation rate in a certain solute concentration range. In this low solute concentration regime, the nucleation barrier is too high to form the nuclei. To sum up, in ambient air, the MA_2_Pb_3_I_8_·2DMSO intermediate nucleates more slowly than it grows, leading to the tendency to form larger and longer fibers in ambient air than in glove box. Clearly, controlling the water assisted nucleation and growth of MA_2_Pb_3_I_8_·2DMSO intermediate to form an optimal density and size of the nuclei is crucial to obtain high quality perovskite films in ambient air.

### The prenucleation strategy for compact film fabrication

To obtain a dense supply of intermediate nuclei for fabricating high-quality perovskite films in ambient air, we developed the prenucleation strategy. Our finding of water induced fast intermediate crystallite growth suggests that the key is to control the concentrations of the lead complexes—the prenucleation clusters—by controlling the solute concentration. Specifically, the process runs as follows. First, a wet film was spun at a slow spinning speed so that the rate of solvent evaporation and water absorption was intentionally kept low. In this way, the solute concentration was allowed to increase to an adequate value but below the nucleation threshold (Supplementary Fig. 6a), so that only a low concentration of prenucleation clusters could be formed. Then, the sample spinning speed was quickly increased to a very high level to form a sufficiently high concentration of prenucleation clusters, that is, the system was swiftly driven to cross the nucleation threshold. Immediately after, the anti-solvent was dripped multiple times onto the wet film to extract solvent in a programmed fashion, which was able to keep the prenucleation cluster concentration above the nucleation threshold. Here the multiple dripping process is pivotal in prolonging the nucleation time: as soon as the prenucleation clusters were consumed to the level close to the nucleation threshold, the next dripping was applied to lift their concentration. Consequently, the significant increase of the nucleation events would result accompanied by the increase of nuclei density (Supplementary Fig. 6b).

The advantage of our prenucleation method in contrast to the conventional solvent dripping method operated in ambient air can be vividly appreciated through videos (Supplementary Movie 1 and 2). Supplementary Movie 1 shows the phenomenon of the conventional solvent dripping process at humidity of 41% and temperature of 27 °C. The solvent dripping procedure was the same as reported in our previous works^10^: we dropped the perovskite precursor on a slide, spun it and then dripped the anti-solvent. After dripping the antisolvent onto the wet film, the film was clearly transparent. Then we annealed this transparent film at 98 °C for 7 min and finally obtained a rough and greyish black perovskite film. For comparison, Supplementary Movie 2 shows the phenomenon of the prenucleation method involved film formation process operated under otherwise the same conditions. The programmed spinning and dripping parameters such as spinning speed and time were finely adjusted to enable abundant formation of the prenucleation clusters, sustained nucleation of the intermediate and smooth conversion to the perovskites (Detailed dripping parameters are provided in the Device Fabrication section). After only two runs of dripping, the wet film turned to light brown and slightly transparent. Then we annealed this slide at the same temperature of 98 °C for 7 min and could obtain a mirror-like and shining black perovskite film, in stark contrast to the transparent film obtained using the conventional solvent dripping method. The two videos prove that the prenucleation method is able to solve the problems intrinsic to the conventional solvent dripping method, especially when operated in ambient air, to obtain high quality perovskite films.

**Fig. 3:**
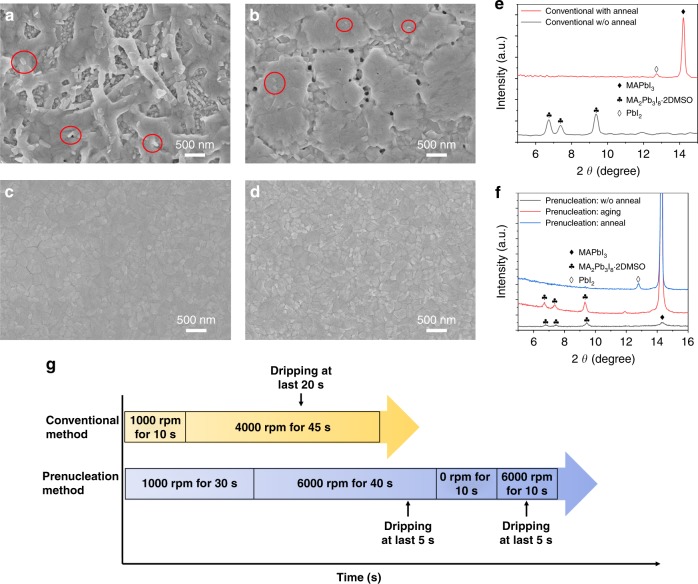
The comparison between conventional and prenucleation methods. The intermediate and perovskite films were synthesized by the spin coating and antisolvent dripping process in ambient atmosphere at 40% RH. The SEM images of **a** the intermediate films without annealing and **b** perovskite films after annealing at 98 °C for 7 min synthesized using the conventional method; the perovskite film is rough and full of pin holes. The SEM images of **c** the intermediate/perovskite films after aging, without annealing and **d** perovskite films after annealing at 98 °C for 7 min synthesized using the prenucleation method; both of the films are compact and smooth. **e** The XRD patterns of the conventional method synthesized intermediate (black curve) and perovskite films (red curve). **f** The XRD patterns of prenucleation method synthesized fresh intermediate (black curve), aged intermediate (red curve) and perovskite films (blue curve). **g** Schematic illustrating the difference between the conventional method and prenucleation methods, the latter being an exquisite programmed spinning and antisolvent dripping process.

Since the videos presented above could only tell the differences in macroscopic phenomenon between the two fabrication methods, we now turn to even finer details, from mesoscopic to nanoscopic, about the different film formation processes in ambient atmosphere. As shown in Fig. 3a, the intermediate film prepared by the conventional solvent dripping method (Fig. 3g) without annealing appeared as a network of rod-shapes structures with the rod’s diameter of about 300 nm and length of about 2 μm. As is well known, the MA_2_Pb_3_I_8_·2DMSO intermediate belongs to the hexagonal crystal system and tends to form a 1D crystal structure, i.e., the nanorods as what the SEM image shows. The XRD pattern further shows that the observed networks are mainly made of the MA_2_Pb_3_I_8_·2DMSO intermediate (Fig. 3e, black curve). Besides, there is a certain amount of smaller rods around 200 nm in length and 30 nm in diameter on the surfaces of the main networks (red circles in Fig. 3a). They might be the nuclei formed in the later periods, which had less time to grow than the bigger ones. Even after the perovskite film was formed by annealing the network film in ambient atmosphere, the morphology still appears as mixed nano-sized blocks and rods (Fig. 3b). Since the MAPbI_3_ perovskite belongs to the tetragonal crystal system and prefers to form crystals with ‘block’ shape as observed, the certain number of abnormal rod-like structures remaining in the film of blocks need to be explained. The XRD pattern shows that the annealed film only contained MAPbI_3_, indicating a nearly full conversion of MA_2_Pb_3_I_8_·2DMSO into MAPbI_3_ with only a small amount of excess PbI_2_. Of note, the MA_2_Pb_3_I_8_·2DMSO intermediate appears as 1 D crystals, whereas the MAPbI_3_ perovskite tends to grow into 3 D crystals, so the conversion from the MA_2_Pb_3_I_8_·2DMSO intermediate into the MAPbI_3_ film requires a significant structural rearrangement. Since the size of MA_2_Pb_3_I_8_·2DMSO obtained from the conventional method is relatively large, its conversion to MAPbI_3_ may partially inherit the rod shape as we just observed.

In contrast, the prenucleation method fabricated films were all of much higher quality. Even without annealing, the prenucleation method (Fig. 3g) yielded a compact and flat film with no rod-like structures as shown in the SEM image in Fig. 3c. The XRD pattern in Fig. 3f (red curve) confirms that the film was mainly made of perovskite with a very small amount of intermediates, and thus is significantly different from the film prepared by the conventional method. Now let’s pay a closer attention to this prenucleation method fabricated film. In ambient atmosphere, the fresh intermediate film made by the prenucleation method without annealing was slightly brown and turned black in 10 min. Supplementary Movie 3 shows this process. The wet film fabricated by prenucleation method was directly removed from spin coater and set aside to age in ambient atmosphere. After several minutes, the film color was changed from transparent to black, clearly due to the aging process. The obtained film was smooth and bright. Since this conversion occurred in only a few min of aging process, comparable to the time needed for the sample transfer from our fabrication lab to SEM lab, we could only observe the mixed intermediate/perovskites phases as shown in Fig. 3c.

To capture the true phases afresh, we acquired XRD patterns of a freshly prepared wet film within 1 min (Fig. 3f, black curve). The film was mainly made of the MA_2_Pb_3_I_8_·2DMSO intermediate mixed with a small amount of MAPbI_3_. During the aging process, the MA_2_Pb_3_I_8_·2DMSO intermediate was largely converted to perovskite, leading to a huge enhancement of the perovskite peak in Fig. 3f, red curve. This conversion process could involve the water assisted formation of MAPbI_3_·H_2_O from the MA_2_Pb_3_I_8_·2DMSO intermediate, which then may be followed by losing a H_2_O molecule to form MAPbI_3_ at room temperature^26,27^. Interestingly, the wet film prepared by our prenucleation method only took several minutes to convert into perovskite (shown in Supplementary Movie 3), whereas as long as 5 h was required in Qiao’s work^17^. While the more antisolvent used in the prenucleation method than in the conventional method has made the film less wet and thus more easily to dry up, the more important cause for the facile intermediate to perovskite conversion should be the smaller size of the intermediate thus formed. As discussed above, the wet films made using the prenucleation method typically consist of uniform crystals with a smaller size than that using the conventional method. One can imagine that the smaller intermediate crystals with a larger surface to volume ratio should be easier to be converted to perovskite than the larger ones due to the smaller activation volume, the shorter escape length of the remaining solvent molecules in the crystals, as well as the more surface exposure to the environment. One example to illustrate the benefit of the more surface exposure to the environment is the interaction with water, which has proved instrumental to the conversion process of MA_2_Pb_3_I_8_·2DMSO intermediate to perovskite^17^. To sum up, the smaller intermediate crystals in the wet films fabricated by prenucleation method shall be easier to be converted to perovskite than the larger intermediate crystals in the wet films fabricated by the conventional method. As a result, the prenucleation method made the wet film transform more easily and more rapidly from the transparent intermediate to the black perovskite even without annealing. Nevertheless, we did anneal the fresh intermediate film for 7 min in ambient atmosphere, and the resulting film morphology was rather similar to that of the aged films (Fig. 3d). The annealing resulted in a more complete conversion to MAPbI_3_ perovskite and, as usual, with a small amount of PbI_2_ mixed in (Fig. 3f, blue curve).

### In situ spectroscopic tracking of nucleation and growth

We firstly identified the characteristic changes of the lead complexes in the UV–vis absorption spectra (Supplementary Fig. 8). The increase of absorption intensity in the wavelength region between 450 nm and 1000 nm indicates formation of the MA_2_Pb_3_I_8_·2DMSO intermediate. The decrease of the 410 nm peak indicates consumption of the PbI_2_·2DMSO and Pb_2_I_4_·2DMSO oligomers or the precursor species. These results provide the basis for using the in-situ UV-vis spectroscopy tests to monitor the precursor wet film formation in the spin-coating process as we will describe below.

To elucidate the film formation process, we performed in-situ UV-vis spectroscopy on the wet precursor films during the spinning coating process following the two different methods. The experimental setup is shown in Supplementary Fig. 10a. The incident light out of an optical fiber passes through the spinning substrate covered with the wet perovskite precursor film, and the attenuated light is then collected by another optical fiber. In this way, we could obtain a series of UV-vis absorption spectra of the wet precursor films at every second during the whole spin-coating process. The results showed that the two different fabrication methods give rise to remarkably different evolutions of the UV-Vis absorption spectra during the spin-coating process. In the simple spinning process without taking the antisolvent dripping procedure, the intensity of absorption peak (wavelength about 400 nm) decreased monotonically and rapidly to zero, while the absorption at the wavelengths above 450 nm increased, indicating the conversion of PbI_2_·2DMSO, Pb_2_I_4_·2DMSO and the precursor species completely to the MA_2_Pb_3_I_8_·2DMSO intermediate after time elapse of over 100 s (Supplementary Fig. 10b). Note that with a time of about 100 s, the size of the MA_2_Pb_3_I_8_·2DMSO intermediate has grown too large to be converted to a high-quality perovskite film (Fig. 2b, which shows the SEM image of this film).

With the conventional method, the peak intensity at 400 nm decreased initially (Supplementary Fig. 10c) and, after the anisole dripping, increased slightly and then decreased slowly. During the whole spin-coating process, however, no obvious absorption increase happened at the wavelengths above 450 nm, indicating little formation of the MA_2_Pb_3_I_8_·2DMSO intermediate. In other words, only a small amount of MA_2_Pb_3_I_8_·2DMSO nuclei formed at the 1^st^ dripping step. After 30 min of aging, however, the absorption peak at 400 nm totally disappeared, while considerable absorption occurred at the wavelengths above 450 nm, indicating the formation of large MA_2_Pb_3_I_8_·2DMSO intermediate crystals with almost no conversion to perovskite (Supplementary Fig. 10d). When we prolonged the spinning time in the conventional method, large MA_2_Pb_3_I_8_·2DMSO intermediate crystals also formed, indicating that this method prefers the formation of only MA_2_Pb_3_I_8_·2DMSO but not perovskite during the spinning process.

Next, with the prenucleation method, the peak intensity at 400 nm decreased quickly at the initial step and increased slightly after the 1^st^ dripping procedure, followed by gradual decrease. However, this peak increased pronouncedly after the 2^nd^ dripping procedure, and remarkable absorption increase happened (above 450 nm) during the following seconds, indicating the abundant formation of the MA_2_Pb_3_I_8_·2DMSO nuclei (Supplementary Fig. 10e). This clearly proves that the prenucleation method crucially changed the intermediate nucleation kinetics over the conventional method by preferentially forming a burst of the PbI_2_·2DMSO and Pb_2_I_4_·2DMSO complexes as prenucleation clusters. In addition, the absorption is also enhanced in the wavelength range between 320 nm to 380 nm characteristic of perovskite, indicating the formation of perovskite nuclei. In stark contrast to the films fabricated by the conventional method, the 30 min aged film fabricated by the prenucleation method showed an enlarged absorption in the wavelength range between 300 to 800 nm, indicating the formation of a large amount of perovskite crystallites (Supplementary Fig. 10f). Taken together, the results presented further support that the prenucleation method enables the formation of abundant (but not too large) MA_2_Pb_3_I_8_·2DMSO nuclei in the precursor film by preferentially creating a burst of the PbI_2_·2DMSO and Pb_2_I_4_·2DMSO complexes as prenucleation clusters. This prenucleation step is crucial for the smooth conversion of abundant (but not too large) MA_2_Pb_3_I_8_·2DMSO nuclei to perovskite while preventing the growth of large intermediate crystals.

### Prenucleation-enabled efficient PVSCs with high consistency

**Fig. 4:**
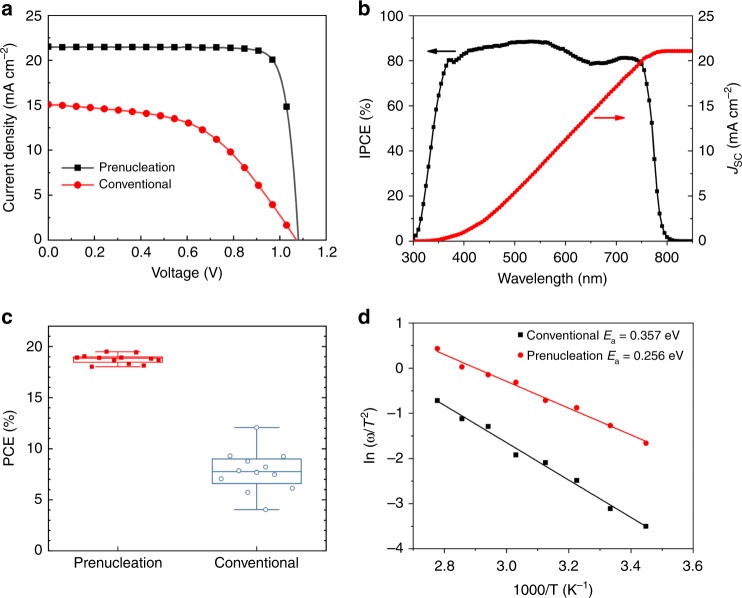
The performance of PVSCs fabricated in ambient atmosphere. **a** Current density versus voltage (*J*-*V*) curves of PVSCs made with perovskite films fabricated using the prenucleation method (black) and the conventional method (red). **b** IPCE curve (black) and integrated current density (*J*_sc_) curve (red) of the PVSC fabricated by the prenucleation method. **c** The statistical data of PVSCs fabricated using the perovskite films prepared by the prenucleation method (red) and the conventional method (blue). In the Box-Whisker Plots, the middle line refers to the median (the second quartile), the box goes from the first quartile to the third quartile, the upper/lower whisker refers to the maximum/minimum values of the data points. **d** The Arrhenius plots of the characteristic transition frequencies of PVSCs fabricated by the prenucleation method (red dots) and conventional method (black squares) and the corresponding calculated trap states energy levels.

Two kinds of perovskite films made by the prenucleation method (prenucleation-PVSC) and the conventional method (convention-PVSC) in ambient atmosphere were used to fabricate solar cells with a p-i-n structure of FTO/NiO_x_/perovskite/PMMA/PCBM/PPDIN6/Ag^19,28^. As expected, the prenucleation-PVSC showed significant improvement in power conversion efficiency (PCE), achieving 19.5% compared to 8.20% of the Convention-PVSC (Fig. 4a). The *J*_sc_ of the prenucleation-PVSC is 21.5 mA cm^−2^, which is much higher than 15.1 mA cm^−2^ of the convention-PVSC. The fill factor (FF) of the prenucleation-PVSC is 0.84, which is much higher than 0.51 of the convention-PVSC. Clearly, the perovskite film quality played the most important role here. Specifically, as the perovskite film of convention-PVSC is rough and full of pin-holes, it is difficult to be uniformly covered by the top electron transport material. In contrast, the perovskite film of Prenucleation-PVSC is compact and pin-hole free, and thus can easily form good and uniform contact to the electron transport material. Consequently, the convention-PVSC should have many more defects and interfacial trap states than the prenucleation-PVSC, leading to slower carrier transport, more back flow electrons and more interfacial recombination, and thus the lower observed *J*_sc_ and FF. On the other hand, the open circuit voltages (*V*_oc_) of the two types of PVSCs are similar. Although it is unclear at present about exactly why the *V*_oc_ values of the two cells are very similar in spite of their large disparity in charge recombination, we notice the much lower fill factor, the much larger series resistance, and the much large ideality factor for the convention-PVSC, which in combination may somehow made the *V*_oc_ comparable to that of the prenucleation-PVSC.

To understand more deeply the origin of the much-improved performance of the prenucleation-PVSC over the convention-PVSC, we performed Time-Resolved Photoluminescence (TRPL) measurements on the perovskite films on glass fabricated by the prenucleation and the conventional methods (Supplementary Fig. 14). The derived carrier life time of the prenucleation-PVK is 87.29 ns, which is twice longer than 44.38 ns for the convention-PVK. The longer lifetime indicates fewer defects induced carrier recombination of the prenucleation-PVK than that of the convention-PVK, in reasonable agreement with the *J–V* curves and the dark current results presented above.

The incident photon to current efficiency (IPCE) curve of optimized PVSC showed a broad plateau above 80% between 380 nm to 760 nm and a sharp decrease in the range between 760 nm to 800 nm, indicating good light utilization of the Prenucleation-PVSCs (Fig. 4b). The corresponding integrated *J*_sc_ from IPCE is 21.1 mA cm^−2^, which agrees well with the value of 21.5 mA cm^−2^ from *J–V* curve (Fig. 4a). In addition, the absorption threshold is 773 nm and the corresponding bandgap is 1.60 eV, calculated by using the half IPCE value point from the IPCE spectrum^29^. In stark contrast, the convention-PVSC has a much lower IPCE plateau value around 60% with an integrated *J*_sc_ of 16.2 mA cm^−2^, again indicating much more severe recombination than the prenucleation-PVSC (Supplementary Fig. 15).

The statistics of the PCE values shows that the performance of prenucleation-PVSCs has very high consistency, with a much narrower distribution than that of convention-PVSCs (Fig. 4c). The champion cell PCE of prenucleation-PVSCs is 19.5% with a *V*_oc_ of 1.08 V, *J*_sc_ of 21.5 mA cm^−2^ and FF of 0.84. The average PCE over 12 devices of prenucleation-PVSCs is 18.8% with the standard deviation of 0.43%. This contrasts sharply with the convention-PVSCs, for which the average PCE is 7.8% and the standard deviation is 1.95%. The significant difference between the two types of devices is again attributed to the different perovskite quality, confirming the high controllability of the prenucleation method. Furthermore, the prenucleation-PVSC shows a decent stability. After 100 days of storage in dry N_2_ filled glove box, the unencapsulated PVSC only lose around 10% of the initial efficiency (Supplementary Fig. 16).

The trap state density and distribution are important properties of the perovskite layers directly related to the recombination behavior of PVSCs. Thus we performed the temperature dependent admittance spectroscopy (TAS) to quantitatively evaluate the trap state density and the trap energy level (*E*_a_)^30^. From our analysis, the *E*_a_ of prenucleation-PVSCs is 0.256 eV, which is smaller than 0.357 eV of convention-PVSCs (Fig. 4d, detailed calculation is in SI part). This means that the trap states in prenucleation-PVSCs is much shallower than those in Convention-PVSCs, leading to a much lower probability of charge recombination for the former. Next, the trap state density of prenucleation-PVSCs is estimated to be 5.49 × 10^15^ cm^−3^, which is only half that of convention-PVSCs (Supplementary Fig. 17b, d). This result again suggests the much lower probability of charge recombination beneficial for achieving a higher efficiency than Convention-PVSCs. In sum, the TAS results provide a reasonable explanation for the superior performance of the cells fabricated by the prenucleation method to those by the conventional method.

**Fig. 5:**
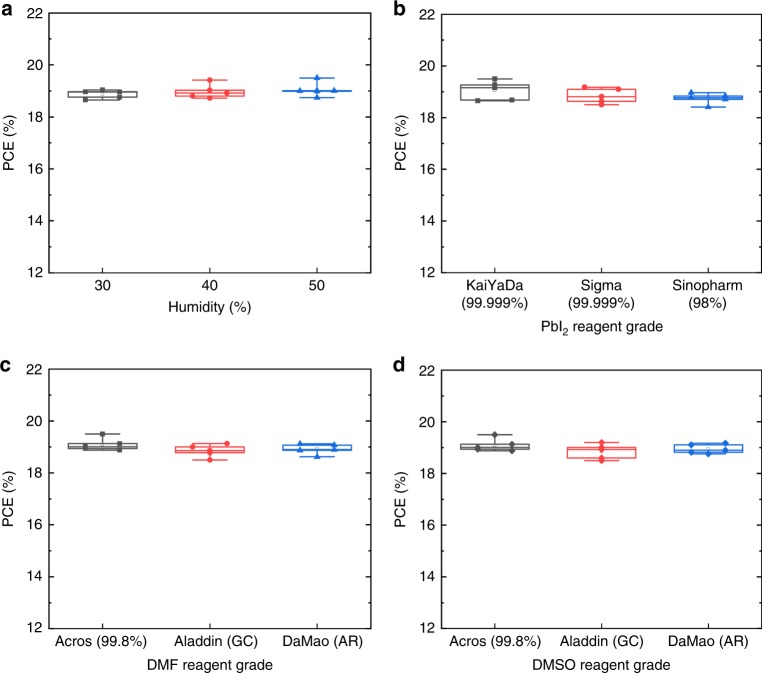
The efficiency distributions of Prenucleation-PVSCs. Devices fabricated **a** at different levels of humidity, **b** using PbI_2_ reagents with different grades, **c** using DMF solvent with different grades and **d** using DMSO solvent with different grades. All the devices showed very similar standard deviations of PCE with the widely different device fabrication conditions and reagents, demonstrating the reproducibility and robustness of the prenucleation method. In the Box-Whisker Plots, the middle line refers to the median (the second quartile), the box goes from the first quartile to the third quartile, the upper/lower whisker refers to the maximum/minimum values of the data points.

To further investigate how the fabrication condition affects the PCEs of prenucleation-PVSCs, we used different reagents to fabricate PVSCs under different humidity. First of all, the PCE values of prenucleation-PVSCs we measured are all around 19% with a standard deviation of only about 0.2%, when the humidity of ambient atmosphere was varied from 30 to 50% (Fig. 5a). This indicates that the prenucleation method is quite robust, and its operation is hardly influenced by the water vapor concentration, a very desirable feature that can largely reduce the cost for environment control in industrial production. Apart from the water vapor, the ambient atmosphere also contains 21% oxygen. However, we found that the oxygen showed little influence on our fabrication process, and high-quality films and high efficiency devices could be easily obtained in the presence of oxygen. It is generally believed that high purity PbI_2_ mixed with organic iodide is required to fabricate high efficiency PVSCs^31^. However, we found that with the prenucleation method, even low purity PbI_2_ (Sinopharm, 98%) could be used to fabricate high-performance PVSCs yielding about 19% PCE with a standard deviation of 0.19% (Fig. 5b). We also tested the effects of impurity from other reagents. As expected, the purity of solvent (DMF or DMSO) showed little influence on the PVSCs performance. Even in the presence of impurities, the DMF or DMSO solvents could still be used to prepare the high-quality intermediate as well as high quality perovskite films. The average PCE of the PVSCs fabricated by the different grade DMF is 18.9% with a standard deviation of only 0.23% (Fig. 5c), and that of the PVSCs by the different grade DMSO is 19.0% with a standard deviation of 0.24% (Fig. 5d). From the purity label, the impurities of chemicals were mainly Fe, which have only weak interactions with the ions in the perovskite precursor solution. Thus, in the conventional method, the impurities in the wet film are expected to gradually precipitate with increasing concentration during the spinning process. In the prenucleation method, however, the lead oligomer intermediates are selectively formed with the assistance of water and precipitate earlier than that in the conventional method, making the former much less sensitive to the impurities. Taken together, the prenucleation method enjoys remarkable advantages, which can enable high performing and consistent PVSCs with a much wider window for fabrication characterized by the tolerance to various reagents and fabrication conditions. This unique advantage is very important and bodes well for the industrial production of PVSCs.

## Discussion

In the present work, we successfully demonstrate a prenucleation method for the fabrication of high-quality perovskite films in ambient atmosphere. The water assisted lead oligomer intermediate formation process is systematically studied through both simulation and experiments, which elucidate the origin of large sized intermediate nanorods formed in ambient atmosphere with the conventional fabrication method. To obtain compact and pinhole-free films, we develop a programmed sample spinning and multiple antisolvent dripping method to continuously form the PbI_2_·2DMSO and Pb_2_I_4_·2DMSO complexes and thus to prolong the nucleation of the intermediates preventing the water-assisted growth into a too large size—the linchpin of the ‘prenucleation method’. Significantly, the quasi-solid intermediate film fabricated by the prenucleation method in ambient atmosphere can be easily converted to a high quality perovskite film by heating or simply aging at room temperature. Impressively, the PVSCs fabricated by the prenucleation method in ambient atmosphere achieve an average PCE of 18.8% with a strikingly small standard deviation of 0.43%, whereas the convention-PVSCs only have an average PCE of 7.8% with a rather large standard deviation of 1.95%. Clearly and most importantly, not only the prenucleation-PVSCs have a much high performance, they are also much more tolerant to the changes in environmental humidity and impurity, exhibiting a superior device yield to the convention-PVSCs. We anticipate that the prenucleation method will largely simplify the industrial fabrication of perovskite films, bringing the commercialization of perovskite materials a significant step closer in photovoltaics and optoelectronics.

## Methods

### Materials

For perovskite precursor solution, besides the anhydrous and high purity reagents, some analytical reagents were compared. The purity degree and impurity content were recorded in brackets. Methylammonium iodide (MAI) was purchased from Dyesol. PbI_2_ reagents were purchased from Sigma-Aldrich (99.999%, trace metals basis), Sinopharm chemical reagent (98%, 0.03% chloride, 0.5% alkaline-earth metals, 0.05% Fe) and Hangzhou Kaiyada (99.999%), respectively. N,N-dimethylformamide (DMF) reagents were purchased from Acros (anhydrous, 99.8%), Aladdin (GC, 99.9%) and Tianjin Damao chemical reagent (AR, 99.5%, 0.005% evaporation residue content, 0.0005% Fe, 0.1% H_2_O), respectively. Dimethyl sulfoxide (DMSO) reagents were purchased from Acros (anhydrous, 99.8%), Aladdin (GC, 99.9%) and Tianjin Damao chemical reagent (AR, 99.5%, 0.02% incinerated residue content, 0.1% H_2_O), respectively. Anisole was purchased from Sigma-Aldrich (anhydrous, 99.98%). Chlorobenzene (CB) was purchased from Acros (extra dry, 99.5%). 2,2,2-Trifluoroethanol (TFE) was purchased from Energy chemical (99.5%). [6,6]-phenyl-C61-butyric acid methyl ester (PC61BM) was purchased from NANO-C Tech. Anisole was purchased from Aladdin (anhydrous, 99.7%). Poly(methyl methacrylate) (PMMA) was purchased from Acros (M.W. 35000).

### Device fabrication

FTO glasses were cleaned using ultrasonic washer in deionized water, ethanol, isopropanol, acetone and ethanol for 30 min sequentially, and dried at 80 °C. NiO_*x*_ layer was prepared using spray method according to our previous paper^28^.

The perovskite precursor solution was modified according to our previous report^19^, briefly, we increase the solution concentration from 1.11 M to 1.79 M. To prepare the 1.79 M precursors solution, 461 mg PbI_2_ and 159 mg MAI were dissolved in 434 μL DMSO and 124 μL DMF. The mixed solution was stirred on 60 °C hotplate overnight. Perovskite film was prepared using the as-prepared solution in a fume hood under the humidity between 20–60%RH. 35 μL perovskite solution was dripped on the NiO_*x*_ substrate. The spin coating process consisted of four steps, which were 1000 rpm for 30 s, 6000 rpm for 40 s, 0 rpm for 10 s, 6000 rpm for 10 s sequentially, as shown in Fig. 3g. During the last 5 s of the second step and the whole process of the fourth step, 80 μL anisole was dripped on the substrate respectively. After the first dripping, the film was still transparent and can maintain the state for long time in ambient air. After the second dripping, the film was transformed into brown, and the as-prepared film was transformed into compact perovskite film at room temperature or 98 °C for 7 min in ambient atmosphere. Different from the conventional fabrication method in dry air glovebox, we intended to prolong the spin time of 1000 rpm and increased the rotate speed of anti-solvent dripping to 6000 rpm, in this way, film thickness and grain uniformity were improved. More importantly, a wider anti-solvent dripping window was achieved using the twice anti-solvent dripping method, so perovskite quality was insensitive to the operating differences and solvent purity. All the control experiment is conducted in dry air glovebox and applied our previous method^28^.

1 mg/mL PMMA was dissolved in CB, and the solution was coated on the perovskite film at 5000 rpm for 30 s. 20 mg/mL PC61BM was dissolved in CB, and the solution was coated on the PMMA film at 3000 rpm for 30 s. 0.5 mg/mL PPDIN6 was dissolved in TFE, and the interface layer was coated on the PCBM film at 3000 rpm for 30 s. The PPDIN6 material was synthesized according to our previous report^30^. Finally, a thickness of 150 nm silver electrode was evaporated on the substrate.

### Device characterization

The morphologies of perovskite were characterized by field emission scanning electron microscope (JEOL 7100 F). The X-ray diffraction (XRD) patterns were obtained using diffractometer (D8 Discover, Bruker AXS, Karlsruhe, Germany) with Cu Kα radiation. The samples were immediately sealed in a valve bag after spin coating, and were measured by diffractometer immediately. UV–vis absorption spectrograms were measured on UV–vis spectrometer (UV-1800, Shimadzu). The steady state PL spectrograms were measured on fluorospectrophotometer (Horiba, FluoroMax-4) using the excitation light source with wavelength of 455 nm. Attenuated Total internal Reflectance Fourier Transform Infrared spectroscopy (ATR-FTIR) spectra were measured on the FT-MIR spectrometers (PerkinElmer Frontier). The current*–*voltage (*J–V*) characteristics of devices were recorded by a Keithley 2400 source meter under 1 Sun (AM 1.5 G, 100 mW cm^−2^, the light intensity was calibrated using a certified standard silicon cell) illumination with a solar simulator (Newport, Oriel Sol3A). The device was tested in nitrogen glovebox at room temperature, in which the contents of water and oxygen are below 1 ppm. The test area was defined by a mask with aperture area of 0.1 cm^2^. The external quantum efficiency (EQE) were measured on the photoelectric detection test system calibrated using a reference silicon cell (Zolix, SCS10-150A-DSSC-CB07).

### Reporting summary

Further information on research design is available in the Nature Research Reporting Summary linked to this article.

## Supplementary information

Supplementary Information

Reporting Summary

Description of Additional Supplementary Files

Supplementary Movie 1

Supplementary Movie 2

Supplementary Movie 3

## Data Availability

The data that support the findings of this study are available from the corresponding author upon request.
