# Supplementary material for: A prenucleation strategy for ambient fabrication of perovskite solar cells with high device performance uniformity
Source: Nat Commun. 2020 Feb 21;11:1006. doi: 10.1038/s41467-020-14715-0 (PMC7035260; doi:10.1038/s41467-020-14715-0)
Supplement: Supplementary file 1 — Supplementary Information [file 41467_2020_14715_MOESM1_ESM.pdf]

A prenucleation strategy for ambient fabrication of perovskite solar cells with high device performance uniformity

Zhang et al.

Supplementary Information

A prenucleation strategy for ambient fabrication of perovskite solar cells with high device performance uniformity

Kai Zhang,[†] Zheng Wang,[†] Gaopeng Wang,[†] Jian Wang,[†] Yu Li[†], Wei Qian,[†] Shizhao Zheng,[†] Shuang Xiao,[†,*] Shihe Yang[†,*]

† Guangdong Provincial Key Lab of Nano-Micro Materials Research, School of Chemical Biology and Biotechnology, Shenzhen Graduate School, Peking University, Shenzhen, China.

KEYWORDS. Perovskite solar cell, ambient fabrication, prenucleation cluster, water-assisted growth, p-i-n structure.

Corresponding Author

* Shuang Xiao, E-mail: xiaoshuang@pku.edu.cn; Shihe Yang, E-mail: chsyang@pku.edu.cn, chsyang@ust.hk;

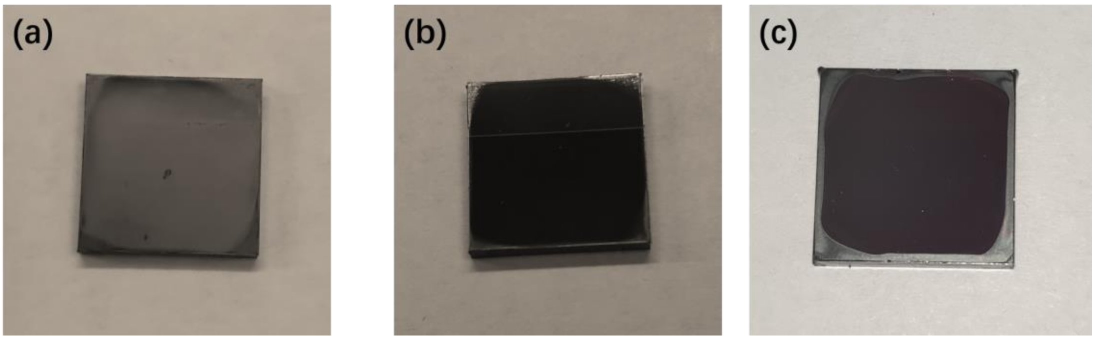

Supplementary Figure 1. The photographs of perovskite films fabricated by (a) conventional method and (b) prenucleation method in ambient air at 40% RH. (c) The perovskite film fabricated by conventional method in dry air glove box.

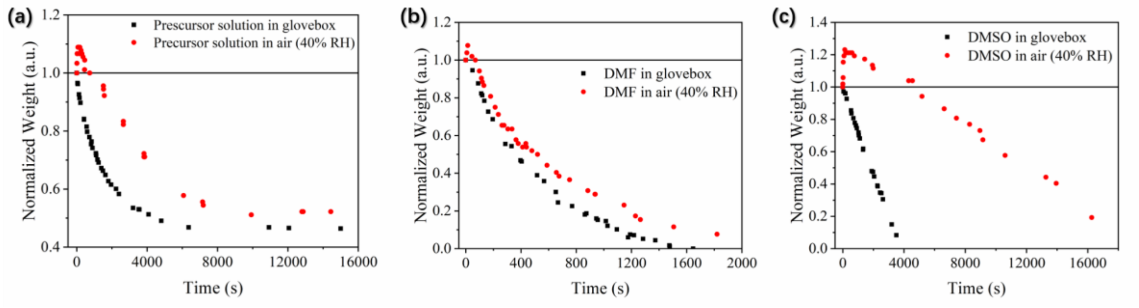

Supplementary Figure 2. The weight change of (a) 1.79 M precursor solution, (b) DMF and (c) DMSO versus time, when exposing in glovebox or in air at 40% RH. The glovebox was filled with N_2 and water concentration was below 1 ppm. For evaluation, the precursor solution, DMF and DMSO were drop casted on glass without spinning process in glovebox or air, respectively. Then, they were weighed by a balance (Sartorius, Secura225D-1CN, Accuracy: 0.02 mg).

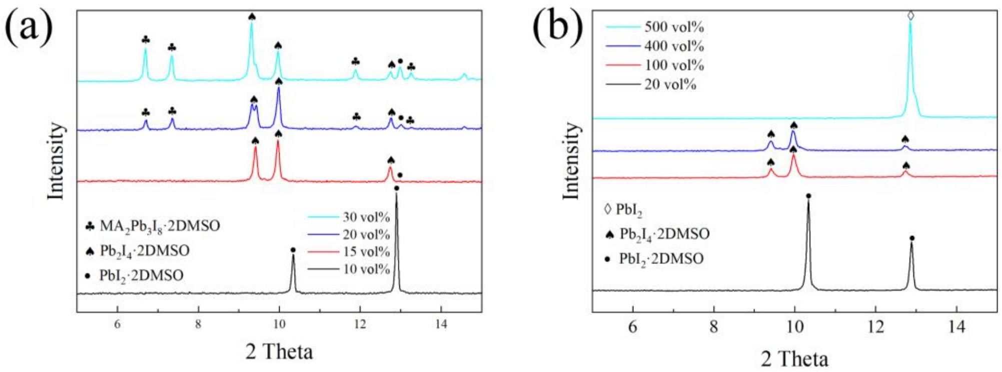

Supplementary Figure 3. XRD patterns of the lead complexes formed by (a) Adding water into perovskite precursor solution, and (b) Adding water into PbI_2/DMSO solution.

H_2O in precursor by volume	Pb1 ($PbI_2 \cdot 2DMSO$)	Pb2 ($Pb_2I_4 \cdot 2DMSO$)	Pb3 ($MA_2Pb_3I_8 \cdot 2DMSO$)
0	×	×	×
10%	√	×	×
15%	√	√	×
20%	√	√	√
30%	√	√	√

Supplementary Table 1. The existence (√) and non-existence (×) of Pb1 ($PbI_2 \cdot 2DMSO$), Pb2 ($Pb_2I_4 \cdot 2DMSO$) and Pb3 ($MA_2Pb_3I_8 \cdot 2DMSO$) cluster crystals in the perovskite precursor with different amount of water added, showing their sequential formation. The result is derived from the XRD patterns in Supplementary Figure 3a. For the precursor without water, it was pure solution and contained no solid crystals. So, it showed no peaks in XRD patterns.

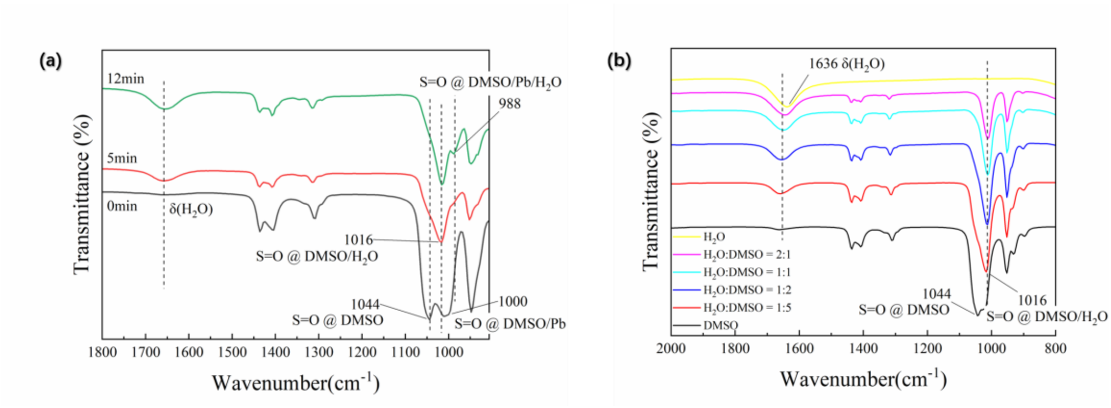

Supplementary Figure 4. Fourier Transform Infrared Spectra (FTIR) of (a) PbI_2 in DMSO solution stored in humid air taken at different time intervals: fresh (black curve), 5 min (red curve) and 12 min (green curve); (b) DMSO/H_2O mixture: DMSO (black curve), H_2O : DMSO = 1:5 (red curve), H_2O : DMSO = 1:2 (blue curve), H_2O : DMSO = 1:1 (light blue curve), H_2O : DMSO = 2:1 (purple curve), H_2O (yellow curve). The FTIR spectra of MAI/PbI_2 in DMSO solution under the same conditions are too complex to obtain reliable conclusions, and thus are not shown here.

Peak Location	Peak Type	Peak information
1636 cm^{-1}	$\delta(H_2O)$	H_2O without interaction
1044 cm^{-1}	$\nu(S{=}O)$	DMSO without interaction
1016 cm^{-1}	$\nu(S{=}O)$	DMSO interacted with H_2O
1000 cm^{-1}	$\nu(S{=}O)$	DMSO interacted with PbI_2
988 cm^{-1}	$\nu(S{=}O)$	DMSO interacted with both H_2O and PbI_2

Supplementary Table 2. Peak assignments of the FTIR spectra. $\delta(H_2O)$ is the bending frequency of H_2O. $\nu(S{=}O)$ is the stretching frequency of S=O. All the FTIR peaks were referenced to the calculation results from the molecules: DMSO, 2DMSO-PbI_2, 2DMSO-PbI_2-H_2O, 2DMSO-2PbI_2 and 2DMSO-2PbI_2-H_2O.

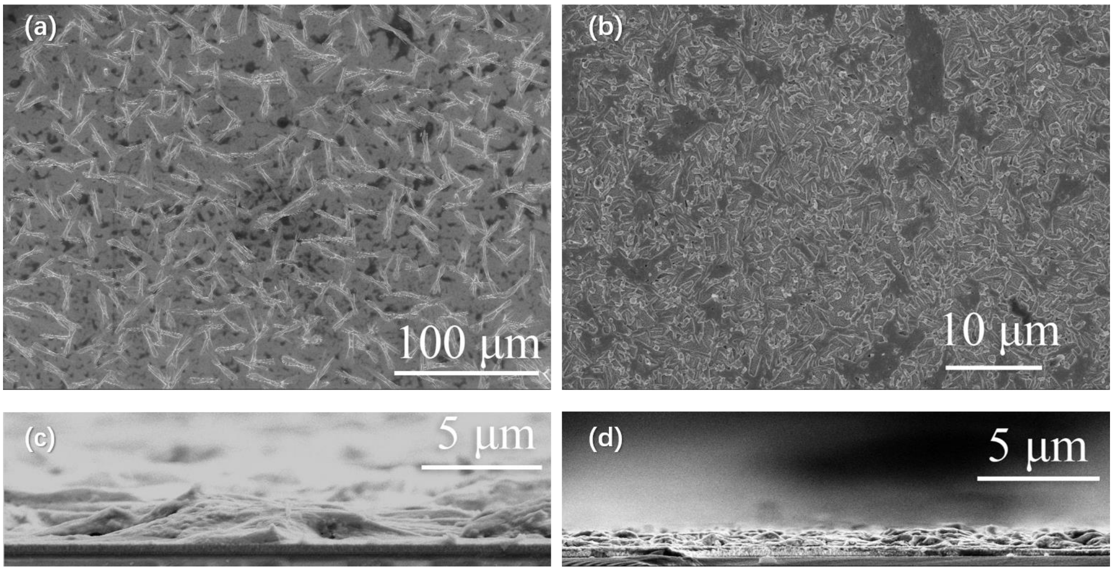

Supplementary Figure 5. The top-view SEM images of the $MA_2Pb_3I_8 \cdot 2DMSO$ intermediate fabricated in (a) ambient atmosphere and (b) glove box. The cross-sectional SEM images of the $MA_2Pb_3I_8 \cdot 2DMSO$ intermediate fabricated in (a) ambient atmosphere and (b) glovebox.

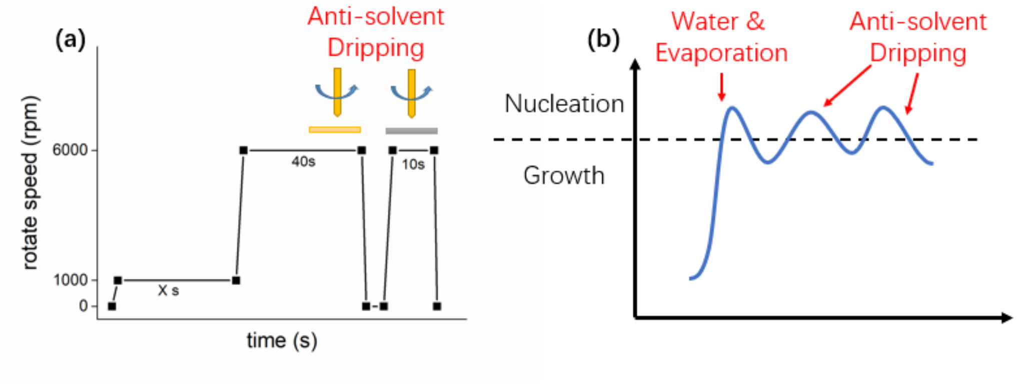

Supplementary Figure 6. (a) The programmed spinning and antisolvent dripping process. (b) Prenucleation scheme for nucleation and growth in the spin-coating process.

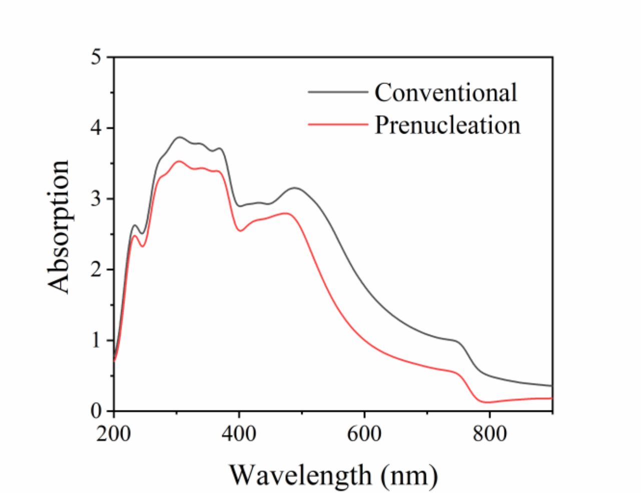

Supplementary Figure 7. The UV-vis spectra of convention-PVSCs (black spectrum), and prenucleation-PVSCs (red spectrum). Both of the $MAPbI_3$ films have the absorption edge near 800 nm. The absorption of convention-PVSCs is stronger than that of prenucleation-PVSCs, because of the strong diffuse reflection of rough film fabricated by conventional method.

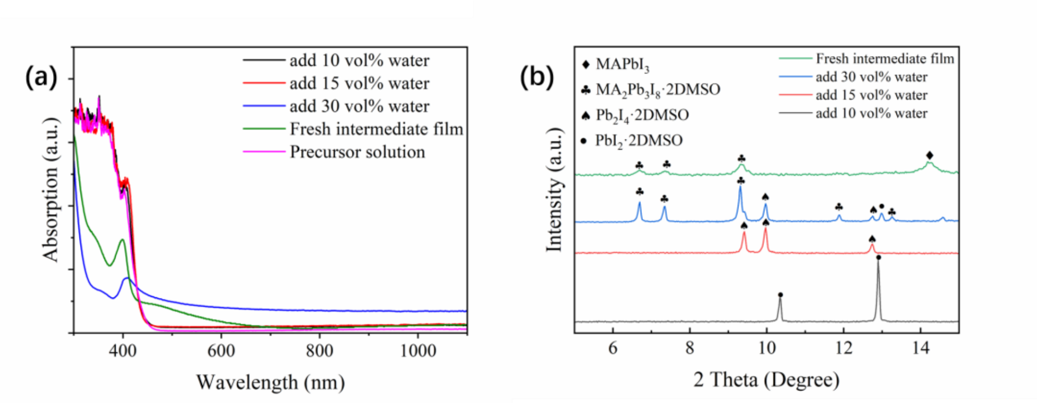

Supplementary Figure 8. (a) UV-vis absorption spectra of the perovskite precursor solution with no water (purple curve), with 10 vol% water added (black curve), 15 vol% water added (red curve) and 30 vol% water added (blue curve), and the fresh intermediate film fabricated by the prenucleation method (green curve). (b) XRD patterns of the perovskite precursor solution with no water (purple curve), with 10 vol% water added (black curve), 15 vol% water added (red curve) and 30 vol% water added (blue curve), and the fresh intermediate film fabricated by the prenucleation method (green curve).

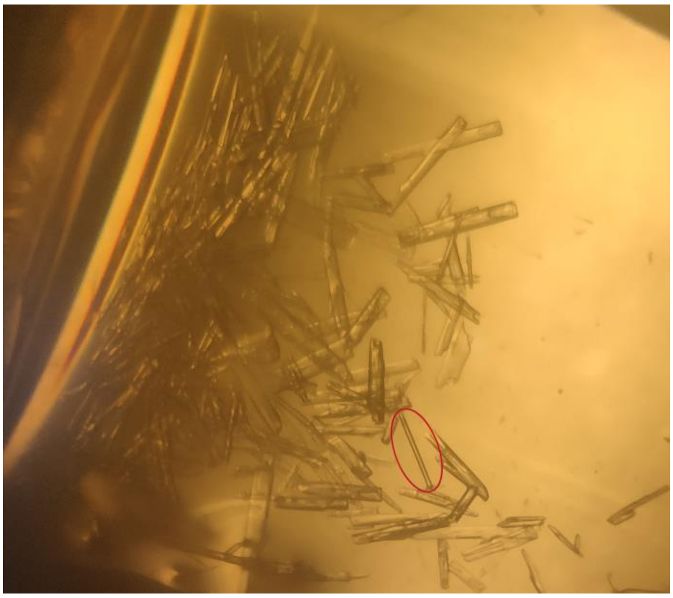

Supplementary Figure 9. The photograph of single crystals of the $PbI_2 \cdot 2DMSO$ complex. Detailed preparation method is described below. The single crystal in the red circle was used for structural analysis.

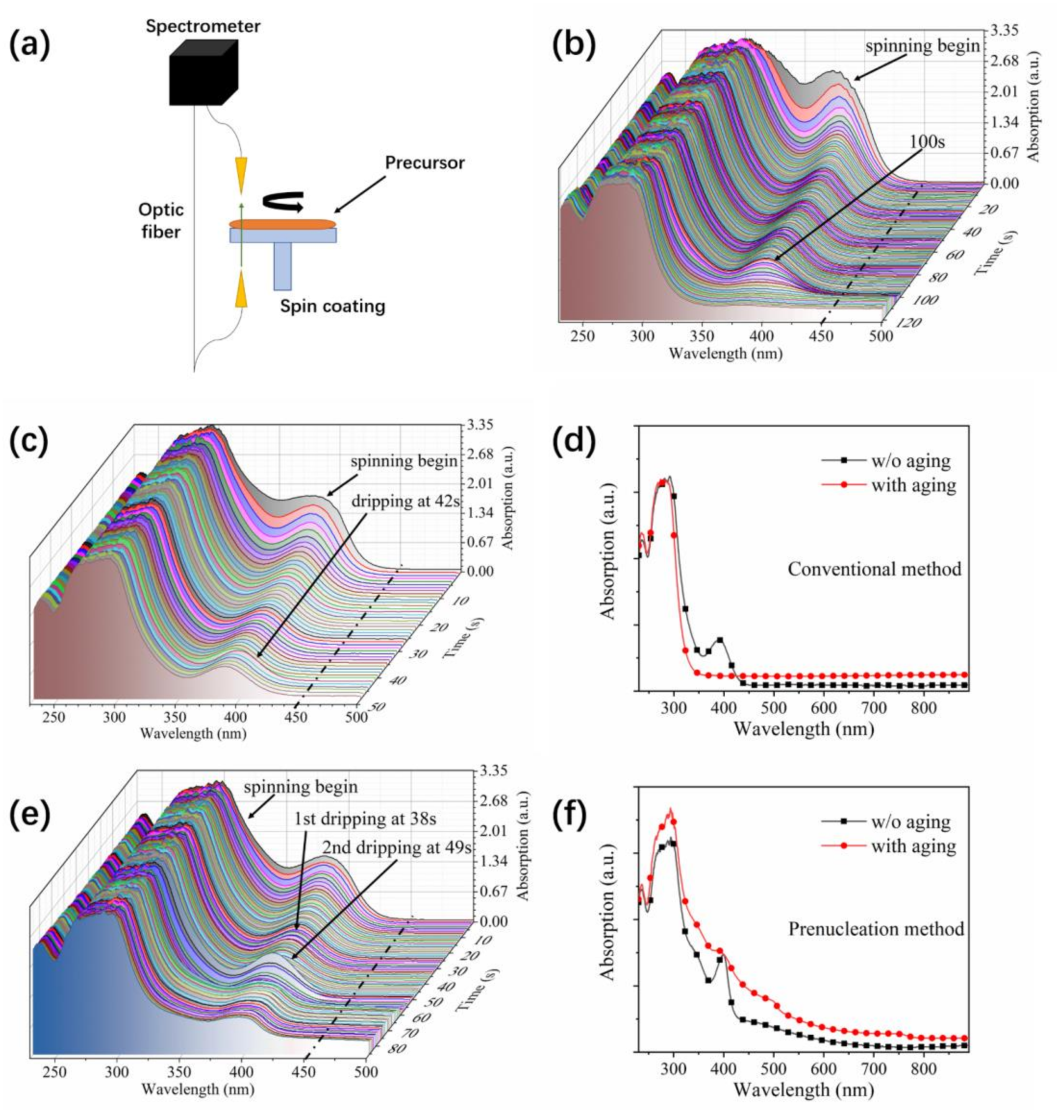

Supplementary Figure 10. The in-situ and ex-situ UV-vis absorption spectroscopy of the evolving wet precursor films in ambient atmosphere (40% RH) on a spinning plate. (a) Schematic of the experimental setup. (b) In-situ UV-vis absorption spectra of the wet precursor film spinning at 6000 rpm for 2 min without taking the antisolvent dripping procedure. (c) In-situ UV-vis absorption spectra of the wet precursor films in the fabrication process following the conventional method. (d) UV-vis absorption spectra of the films fabricated by the conventional method with (red) or without (black) the 30 min aging procedure. (e) In-situ UV-vis absorption spectra of the wet precursor films in the fabrication process following the prenucleation method. (f) UV-vis absorption spectra of films fabricated by the prenucleation method with (red) or without (black) the 30 min aging procedure.

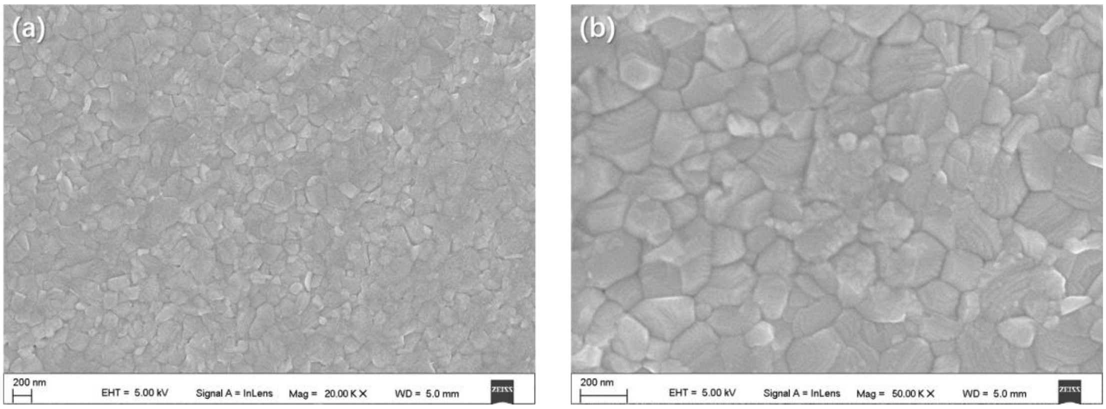

Supplementary Figure 11. The SEM images of a perovskite film fabricated by the conventional method in N_2 filled glovebox: (a) magnification = 20 KX, (b) magnification = 50 KX.

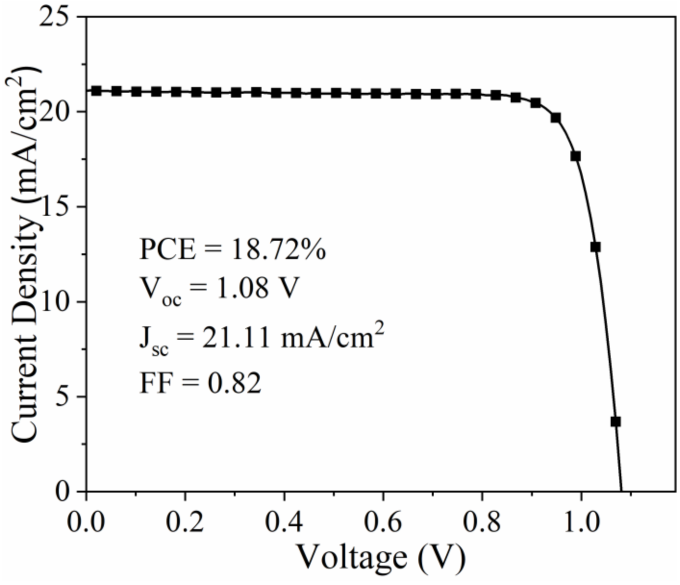

Supplementary Figure 12. The *J*-*V* curve of the PVSCs fabricated by the conventional method in N_2 filled glovebox with PCE of 18.72%, V_{oc} of 1.08 V, J_{sc} of 21.11 mA cm^{-2} and FF of 0.82.

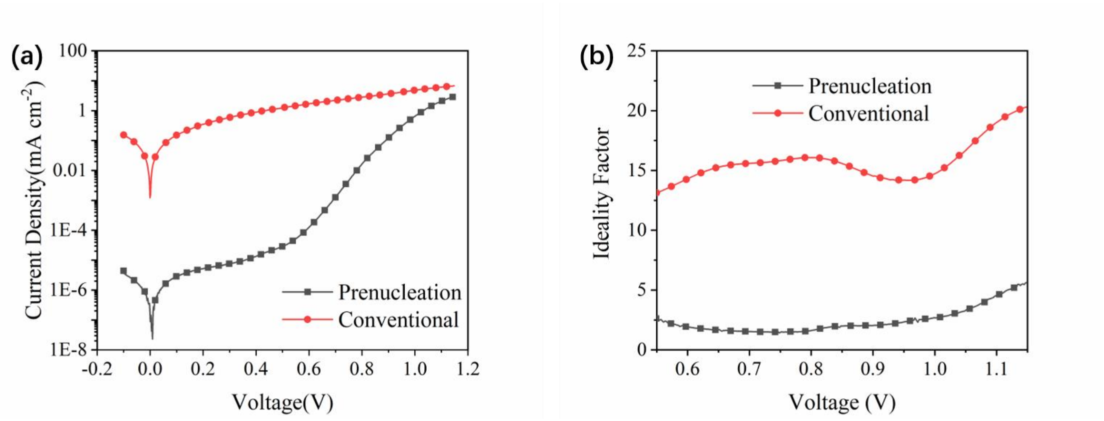

Supplementary Figure 13. (a) Dark current versus voltage curve of prenucleation-PVSC (Black) and convention-PVSC (Red). (b) The ideality factor versus voltage curve of prenucleation-PVSC (Black) and convention-PVSC (Red).

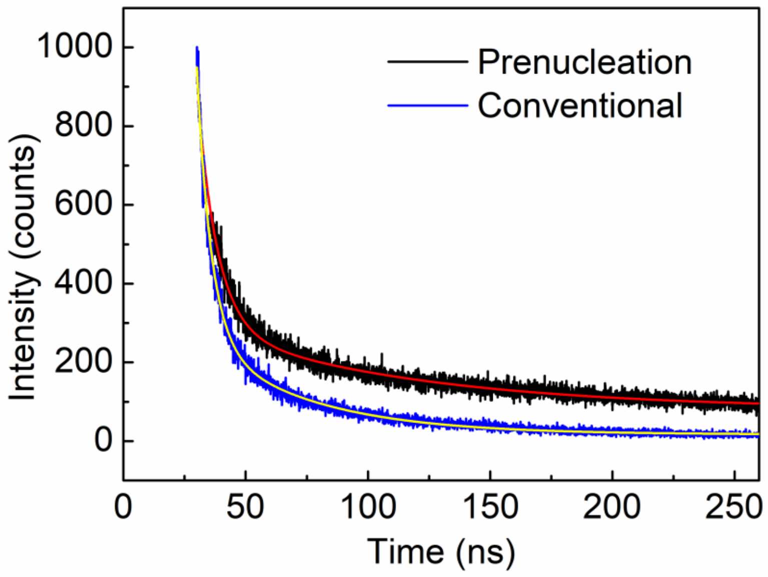

Supplementary Figure 14. The time-resolved photoluminescence curves of the $MAPbI_3$ films fabricated by prenucleation method and conventional method. The perovskite film is directly fabricated on bare glass substrate.

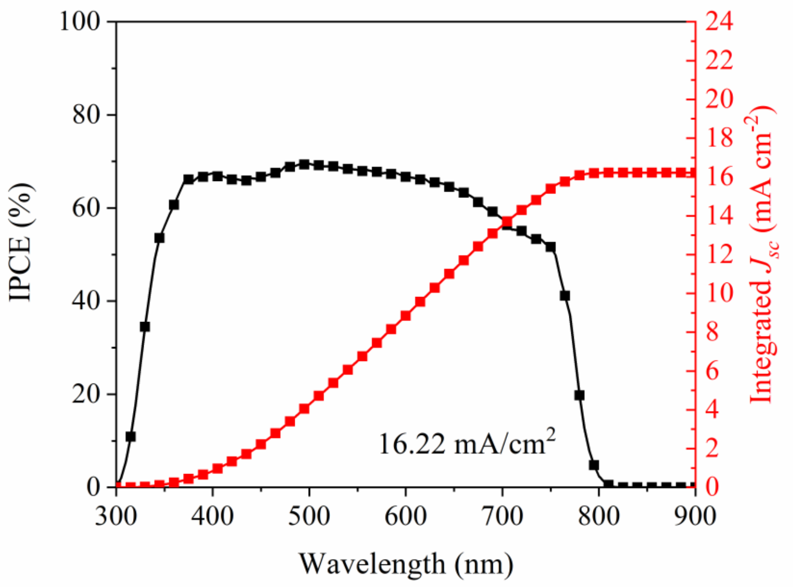

Supplementary Figure 15. The IPCE curve (black) and integrated current density (J_{sc}) curve (red) of the PVSC fabricated by the conventional method.

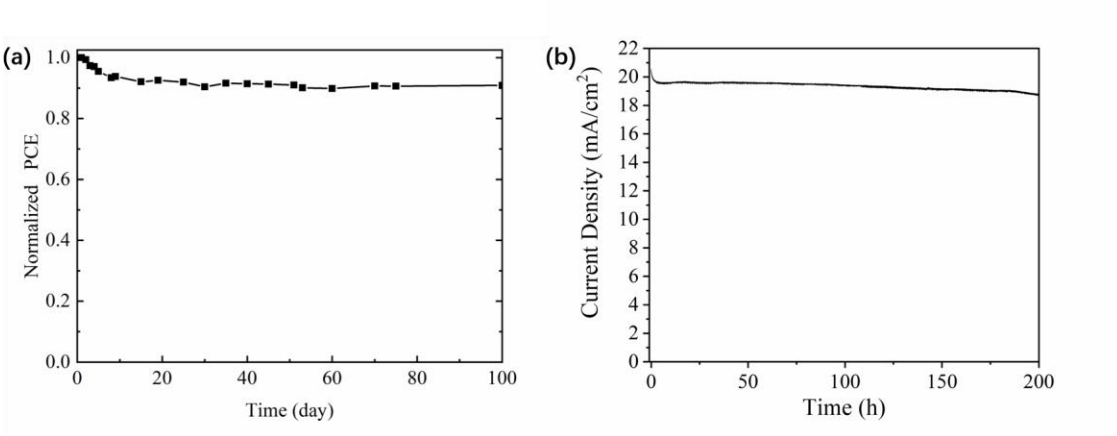

Supplementary Figure 16. (a) The long-term stability of Prenucleation-PVSCs without encapsulation in N_2 filled glove box. (b) The stabilized maximum power point tracking (MPPT), in a N_2 filled glovebox, of the unencapsulated PVSC fabricated by the ambient prenucleation method. The main reason for the initial fast decay may be due to ion migration.[6-8]

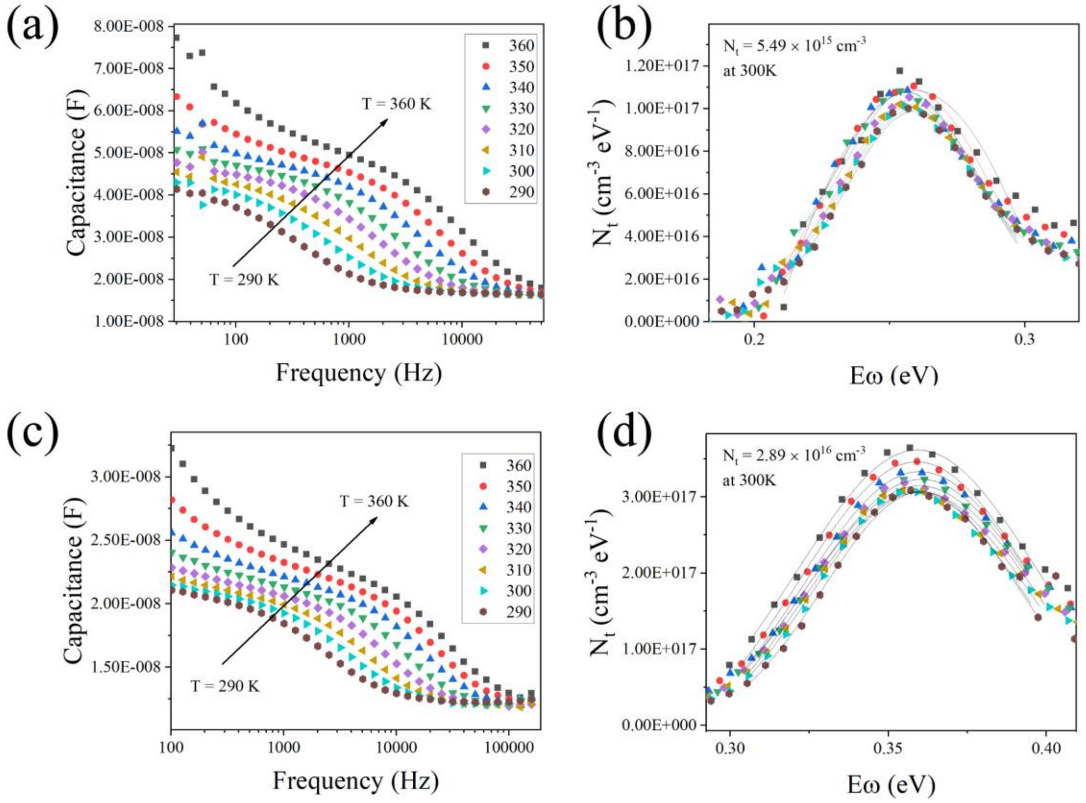

Supplementary Figure 17. Temperature dependent capacitance spectra of (a) convention-PVSCs, (c) prenucleation-PVSCs. The energetic trap distributions of (b) convention-PVSCs, (d) prenucleation-PVSCs.

Supplementary Notes

Computational Details.

The calculation details of the electronic energies of intermediate phases are: All the density functional theory (DFT) calculations were performed using Vienna ab initio simulation package (VASP) [1,2]. The generalized gradient approximation with the Perdew, Burke, and Ernzerhof (PBE) functional[3] was used to describe the electronic exchange and correlation. While the core-valance interactions were modeled by projector augmented wave (PAW) potentials [4]. The energy cutoff for the plane-wave basis set is set as 500 eV. The Brillouin zone was sampled by (9 * 3 * 1) Monkhorst–Pack k-point mesh. And the Broyden method was employed for geometry relaxation until the maximal forces on each relaxed atom were less than 0.01 eV/Å as well as the relative energies were converged within 10^{-5} eV. All the structures are shown by VESTA with 4 unit-cells along the chain of lead oligomeric intermediate [5].

The Evaporation and Water Absorption of Precursor Solution and Solvents.

Weight of all the precursor, DMF and DMSO increased at the initial stage when exposed in humid air (Supplementary Figure 2). In contrast, the weight of precursor, DMF and DMSO decreased monotonously when stored in glovebox. These results indicate that the precursor indeed absorbed water vapor in humid air, while the weight decrease in glovebox was due to the solvent evaporation.

Preparation of Lead Complex from Precursor Solution.

To prepare 2.34 M perovskite precursor, 461 mg PbI_2 and 159 mg MAI were dissolved in 333 μL DMSO and 95 μL DMF. The solution was stirred on 60°C hotplate overnight. To prepare PbI_2 solution, 1 mmol of PbI_2 was dissolved in 700 μL of DMSO. The solution was also stirred on 60 ^{o}C hotplate overnight. In order to avoid

the influence of atmospheric moisture, the XRD samples were measured in a small valve bag (4x6 cm^2). For the samples containing water, H_2O and above solution were quickly added into the valve bag with a fixed volume of 50 μL.

Water Assisted Pb Intermediates Evolution in Precursor Solution.

By adding 10 vol% water into the precursor solution, only Pb1 was formed, in agreement with the water-assisted initial formation of Pb1 as shown in Figure 1. When the added water amount was increased to 15 vol%, abundant Pb2 was formed at the expense of the Pb1, again in agreement with the water assisted Pb1 to Pb2 dimerization process as shown in Figure 1. By increasing the added water amount to 20 vol%, Pb3 was also formed accompanied by the decrease of the Pb2, signifying the water assisted Pb2 to Pb3 sequential clusterization process in Figure 1. However, further adding water up to 30 vol% only resulted in more Pb3 formation instead of the formation of higher cluster crystals, which also agrees with the mechanistic picture depicted in Figure 1. Overall, these results strongly support our simulation results of precursor solution evolution process.

Preparation and Analysis of Single Crystals.

200 μL of an as-prepared perovskite precursor solution was put in a 2 mL unsealed bottle, and the bottle was then placed in a room with relative humidity of 40-60%. Due to the strong hygroscopic property of DMSO, water vapor in ambient air would gradually diffuse into the precursor solution to promote the growth of prenucleation clusters. After 2 to 3 days, needle-like crystals grew in the solution into large sizes. Afterwards, the bottle was sealed and transformed into glovebox, and high-quality single crystals were picked up under the optical microscope. The mother solution residue on the crystal surface was removed by repeated adsorption of glass slide. The crystals were then coated by vacuum grease and incised into a moderate size for structural analysis. The chosen crystal was put into a single crystal diffractometer, and diffraction images were collected using Cu Kα (λ=1.5419 Å) radiation.

Crystallographic analysis and refinement were performed using SHELXS and SHELXL, respectively. The positions of the non-hydrogen atoms were obtained by differential Fourier transform method, and the positions of the hydrogen atoms were obtained by theoretical hydrogenation. The obtained structure was refined by the full least-squares method.

In-situ UV-Vis Spectroscopy.

UV-vis absorption spectra and XRD patterns of the perovskite precursor solutions with different amount of water, as well as the fresh intermediate films prepared by the prenucleation method were acquired and are shown in Supplementary Figure 8 side by side to clarify the characteristic absorption peaks of the in-situ formed lead complexes, namely the $PbI_2 \cdot 2DMSO$, $Pb_2I_4 \cdot 2DMSO$ and $MA_2Pb_3I_8 \cdot 2DMSO$ complexes. We also acquired single crystal diffraction data of the $PbI_2 \cdot 2DMSO$ complex and authenticated its atomic structure (Supplementary Figure 9). The precursor solution exhibits a large absorption peak at ~ 410 nm (Supplementary Figure 8a, purple curve). After adding 10 vol% water into the precursor solution, the $PbI_2 \cdot 2DMSO$ complex was formed, while the absorption peak remained nearly unchanged (Supplementary Figure 8a, black curve / Supplementary Figure 8b, black curve), suggesting insensitivity of the absorption to the change in coordination structure to $PbI_2 \cdot 2DMSO$ from the precursor solution. This remained the case even after 15 vol% water was added into the precursor solution, wherein the $Pb_2I_4 \cdot 2DMSO$ complex was formed (Supplementary Figure 8a, red curve / Supplementary Figure 8b, red curve). After adding 30 vol% water into the precursor solution, however, the $MA_2Pb_3I_8 \cdot 2DMSO$ intermediate was formed in addition to $PbI_2 \cdot 2DMSO$ and $Pb_2I_4 \cdot 2DMSO$ (Supplementary Figure 8b, blue curve), resulting in substantial absorption in the range between 450 nm and 1000 nm at the expense of reduced absorption in the range between 300 nm and 450 nm (Supplementary Figure 8a, blue curve). These spectral features between 450 nm and 1000 nm allowed us to identify the formation process of the $MA_2Pb_3I_8 \cdot 2DMSO$ intermediate. Besides, the decreased

410 nm peak indicates the consumption of the $PbI_2 \cdot 2DMSO$ and $Pb_2I_4 \cdot 2DMSO$ oligomers or the precursor species. These results provide the basis for using the in-situ UV-vis spectroscopy tests to monitor the precursor wet film formation in the spin-coating process.

The Correlation between the Processing Steps, Morphology and Device Operation.

Recall that the film fabricated by the conventional method in humid air is rough and full of pin-holes (see Figure 3b in the main paper). In marked contrast, the film fabricated by prenucleation method in humid air is smooth and pin-hole free (see Figure 3d in the main paper). Expectedly, the perovskite film fabricated by the conventional method in glovebox is also dense and pin-hole free (Supplementary Figure 11), similar to the perovskite film fabricated by the prenucleation method in humid air.

The power conversion efficiencies (PCE) of PVSCs with perovskite films fabricated by the prenucleation method in humid air, the conventional method in glovebox, and the conventional method in humid air are 19.5%, 18.7% and 8.2%, respectively (Figure 4a & Supplementary Figure 12), demonstrating the good correlation between film processing, film morphology and device efficiency. Taken together, the conclusions are:

1) Highest quality perovskite films could be obtained by the prenucleation method in humid air, and high-quality perovskite films could also be obtained by the conventional method in glovebox. These two types of perovskite films yielded the highest and high-performance solar cells, respectively.
2) Low quality perovskite films were obtained by the conventional method in humid air, yielding the poor performance solar cells.

Thus, the good correlation of between the processing, morphology and device operational is experimentally established by comparing the above 3 types of

perovskite films and PVSCs.

Ideality Factor Analysis.

The steep rise of the dark current of PVSCs results from the diffusion dominated current, which can be described by the Shockley diode equation:

$$J = J_0[e^{\frac{qV}{nkT}} - 1] \qquad (1)$$

Where n is the ideality factor, k is the Boltzmann constant, T is the temperature, q is the charge of an electron, V is the voltage and J_0 is the saturated reverse current. By fitting the data to this equation, we could derive ideality factor and saturated reverse current.

As shown in Supplementary Figure 13a, the dark current vs. voltage curves exhibit a much lower leakage current for the prenucleation-PVSC than that of the convention-PVSC, indicating a larger undesirable back-flow of carriers in convention-PVSC. The ideality factor n of convention-PVSCs is around 15, much larger than that of prenucleation-PVSCs (1.6) (Supplementary Figure 13b). Meanwhile, the saturated reverse current J_0 of convention-PVSCs is around 0.342 mA cm^{-2}, again much larger than that of prenucleation-PVSCs (4.3×10^{-7} mA cm^{-2}). For an ideal diode, the ideality factor is equal to 1, and a larger n value commonly indicates more recombination inside the diode. It follows that the much larger ideality factor of the convention-PVSC points to the much more serious non-radiative recombination in the device than in the prenucleation PVSCs. In a solar cell, the saturated reverse current mainly consists of leakage and recombination currents. A larger J_0 value of convention-PVSCs indicates larger leakage and recombination, which leads a lower PCE.

The Analysis of Time-Resolved Photoluminescence (TRPL).

TRPL was performed on the PicoFluo TRPL system (model: Pico-1000). The wavelength of excitation light and detection light were 405 nm and 767 nm, respectively. The sample structure is $MAPbI_3$ on bare glass. For this structure, we applied a model with 2 typical decay routes: (1) the fast non-radiative decay route from defects mediated recombination at $MAPbI_3$/glass, bare $MAPbI_3$ and so on; (2) the slow radiative decay route from direct recombination of electrons in conduction band and holes in valence band. The formula is:

$$y = y_0 + A_1 e^{-x/t_1} + A_2 e^{-x/t_2} \qquad (2)$$

Where the t_2 refer to the life time of carriers.

The Analysis of Temperature dependent Admittance Spectroscopy (TAS).

TAS was measured on Zahner Electrochemical Workstation in the dark from 1 to 10^6 Hz, equipped with a liquid nitrogen cryostat (Oxford Instruments). Both devices had a geometry of FTO/NiO_x/$MAPbI_3$/PMMA/PCBM/PPDIN6/Ag. The temperatures were changed from 290 K to 360 K, and each of temperature stayed for 30min to record the spectrums. The AC amplitude was set as 10 mV, and DC bias was set as 0 mV. The trap energy level (E_a) was obtained via the following equation:

$$\omega_0 = \beta T^2 exp\left(\frac{-E_a}{kT}\right) \qquad (3)$$

where the characteristic transition angular frequency (ω_0) was obtained from derivative of the capacitance–frequency spectrum, β is temperature-independent parameter, k is the Boltzmann constant, and T is temperature, respectively. The trap density (N_t) was obtained according to the following equations:

$$N_t(E_\omega) = -\frac{V_{bi}}{qW}\frac{dC}{d\omega}\frac{\omega}{kT} \qquad (4)$$

$$E_\omega = kTln\frac{2\beta_c N}{\omega} \qquad (5)$$

In the equation (4), V_{bi} is the buil-in potential and W is depletion width, which were obtained from Mott–Schottky analysis, and q is the elementary charge. In the equation (5), β_c is capture coefficient, which is temperature independent.

The deeper traps increasingly participate in transitions as the frequency decreases, and the capacitance increases with temperature in the low frequency region (100 to 10000 Hz). We extracted the characteristic transition frequency (ω_0) from the peak of [$-\omega \times dC/d\omega$] curve, and ω_0 decreases with temperature. The trap energy level was obtained from the fitting curve of $ln(\omega_0/T^2)$ *vs. 1/T*.

In solar cells, the trap density of states and their effects are related to junction capacitance, which are hard to be observed and quantified[11]. Temperature dependent admittance spectroscopy (TAS) on complete devices is commonly used to extract the distribution of trap density and the energetic position of the traps within the band gap by tracing the junction capacitance[11-15], For a p-type perovskite semiconductor, the trap activation energy (E_a) is approximately the depth of trap states energy level relative to the valance band of the perovskite[13].

In our study, both devices had a same geometry and were prepared in the same batch using the same conditions. The only difference is the perovskite layers which were prepared using the conventional and pre-nucleation methods, respectively. Therefore, the influence of interfaces and contacts should be minimized. Thereafter, the difference of measured trap density of states should be attributed to the bulk.

Supplementary References:

1. Kresse, G. & Furthmüller, J. Efficiency of ab-initio total energy calculations for metals and semiconductors using a plane-wave basis set. Comput. Mater. Sci. 6, 15-50 (1996).

2. Kresse, G. & Furthmüller, J. Efficient iterative schemes for ab initio total-energy calculations using a plane-wave basis set. Phys. Rev. B 54, 11169-11186 (1996).

3. Perdew, J. P., Burke, K. & Ernzerhof, M. Generalized gradient approximation made simple. Phys. Rev. Lett. 77, 3865-3868 (1996).

4. Blöchl, P. E. Projector augmented-wave method. Phys. Rev. B 50, 17953-17979 (1994).

5. Momma, K. & Izumi, F. VESTA 3 for three-dimensional visualization of crystal, volumetric and morphology data. J. Appl. Crystallogr. 44, 1272-1276 (2011).

6. Bai, Y. *et al.* A pure and stable intermediate phase is key to growing aligned and vertically monolithic perovskite crystals for efficient PIN planar perovskite solar cells with high processibility and stability. Nano Energy 34, 58–68 (2017).

7. Domanski, K. et al. Not All That Glitters Is Gold: Metal-Migration Induced Degradation in Perovskite Solar Cells. ACS NANO 10, 6306−6314 (2016).

8. Christians, J.A., Schulz, P., Tinkham, J.S. et al. Tailored interfaces of unencapsulated perovskite solar cells for >1,000 hour operational stability. Nat. Energy 3, 68–74 (2018)

9. Zhou X, et al. Role of Moisture in the Preparation of Efficient Planar Perovskite Solar Cells. ACS Sustain. Chem. Eng. 7, 17691-17696 (2019).

10. Gan Z, et al. The Dominant Energy Transport Pathway in Halide Perovskites: Photon Recycling or Carrier Diffusion? Adv. Energy Mater. 9, 1900185 (2019).

11. Duan HS, et al. The identification and characterization of defect states in hybrid organic-inorganic perovskite photovoltaics. Phys. Chem. Chem. Phys. 17, 112-116 (2015).

12. Walter T, Herberholz R, Muller C, Schock HW. Determination of defect

distributions from admittance measurements and application to Cu(In,Ga)Se-2 based heterojunctions. J. Appl. Phys. 80, 4411-4420 (1996).

13. Chen YH, et al. Impacts of alkaline on the defects property and crystallization kinetics in perovskite solar cells. Nat. Commun. 10, 1-10 (2019).

14. Ball JM, Petrozza A. Defects in perovskite-halides and their effects in solar cells. Nat. Energy 1, 1-13 (2016).

15. Yang S, et al. Stabilizing halide perovskite surfaces for solar cell operation with wide-bandgap lead oxysalts. Science 365, 473-478 (2019).
