# Supplementary material for: A prenucleation strategy for ambient fabrication of perovskite solar cells with high device performance uniformity
Source: Nat Commun. 2020 Feb 21;11:1006. doi: 10.1038/s41467-020-14715-0 (PMC7035260; doi:10.1038/s41467-020-14715-0)
Supplement: Supplementary file 2 — Reporting Summary [file 41467_2020_14715_MOESM2_ESM.pdf]

natureresearch

Corresponding author(s): Shihe Yang

Solar Cells Reporting Summary

Nature Research wishes to improve the reproducibility of the work that we publish. This form is intended for publication with all accepted papers reporting the characterization of photovoltaic devices and provides structure for consistency and transparency in reporting. Some list items might not apply to an individual manuscript, but all fields must be completed for clarity.

For further information on Nature Research policies, including our data availability policy, see Authors & Referees.

ü Experimental design

Please check: are the following details reported in the manuscript?

1. Dimensions

Area of the tested solar cells
- [x] Yes
- [ ] No

The test area can be found in the section of Device Characterization.

Method used to determine the device area
- [x] Yes
- [ ] No

The method can be found in the section of Device Characterization.

2. Current-voltage characterization

Current density-voltage (J-V) plots in both forward and backward direction
- [ ] Yes
- [x] No

The hysteresis is very low in our device, and there is very small difference for the forward and backward scan direction. So, we only put the J-V plots in backward direction.

Voltage scan conditions
For instance: scan direction, speed, dwell times
- [ ] Yes
- [x] No

We test device using a common scan conditions with speed of 100mV/s,dwell time of 100ms, backward and forward direction. In this work, the hysteresis is low, and we test the device using same scan conditions. So, we do not place the scan conditions into the text.

Test environment
For instance: characterization temperature, in air or in glove box
- [x] Yes
- [ ] No

The test environment can be found in the section of Device Characterization.

Protocol for preconditioning of the device before its characterization
- [x] Yes
- [ ] No

The protocol for preconditioning of the device can be found in the section of Device Characterization and Supplementary Figure 16.

Stability of the J-V characteristic
Verified with time evolution of the maximum power point or with the photocurrent at maximum power point; see ref. 7 for details.
- [x] Yes
- [ ] No

The stability of the the J-V characteristic can be found in Supplementary Figure 16.

3. Hysteresis or any other unusual behaviour

Description of the unusual behaviour observed during the characterization
- [ ] Yes
- [x] No

There are not unusual behaviour observed during the characterization. The device has low hysteresis and long stability.

Related experimental data
- [x] Yes
- [ ] No

The related experimental data can be found in Figure 4, Figure 5 and Supplementary Figure 16.

4. Efficiency

External quantum efficiency (EQE) or incident photons to current efficiency (IPCE)
- [x] Yes
- [ ] No

The IPCE can be found in Figure 4 and Supplementary Figure 15.

A comparison between the integrated response under the standard reference spectrum and the response measure under the simulator
- [x] Yes
- [ ] No

The comparison can be found in Figure 4 and the section of Prenucleation-enabled Efficient PVSC with High Consistency.

For tandem solar cells, the bias illumination and bias voltage used for each subcell
- [ ] Yes
- [x] No

This work does not contain the tandem solar cells.

5. Calibration

Light source and reference cell or sensor used for the characterization
- [x] Yes
- [ ] No

The calibration can be found in section of Device Characterization.

Confirmation that the reference cell was calibrated and certified

- [x] Yes
- [ ] No

This information can be found in section of Device Characterization.

Calculation of spectral mismatch between the reference cell and the devices under test

- [ ] Yes
- [x] No

We use the certified standard silicon cell as a reference cell. Altough there are some spectral mismatches, the silicon cell absorbs wider spectrum than perovskite solar cell and has long stability, which can guarantee light intensity almost same under ever test.

6. Mask/aperture

Size of the mask/aperture used during testing

- [x] Yes
- [ ] No

The size of the mask/aperture can be found in section of Device Characterization.

Variation of the measured short-circuit current density with the mask/aperture area

- [ ] Yes
- [x] No

In this work, we do not compare the variation of Jsc with the aperture area. The active area of our device is 0.15cm2, and the aperture area of mask is 0.1 cm2.

7. Performance certification

Identity of the independent certification laboratory that confirmed the photovoltaic performance

- [ ] Yes
- [x] No

In this work, we do not carry out a performance certification in independent certification laboratory.

A copy of any certificate(s)
Provide in Supplementary Information

- [ ] Yes
- [x] No

In this work, we do not carry out a performance certification in independent certification laboratory.

8. Statistics

Number of solar cells tested

- [x] Yes
- [ ] No

The statistics number of solar cells can be found in Figure 4.

Statistical analysis of the device performance

- [x] Yes
- [ ] No

The statistical analysis of the device performance can be found in Figure 4 and the section of Prenucleation-enabled Efficient PVSC with High Consistency..

9. Long-term stability analysis

Type of analysis, bias conditions and environmental conditions
For instance: illumination type, temperature, atmosphere humidity, encapsulation method, preconditioning temperature

- [x] Yes
- [ ] No

Long-term stability analysis can be found in Supplementary Figure 16.
