# Supplementary material for: A prenucleation strategy for ambient fabrication of perovskite solar cells with high device performance uniformity
Source: Nat Commun. 2020 Feb 21;11:1006. doi: 10.1038/s41467-020-14715-0 (PMC7035260; doi:10.1038/s41467-020-14715-0)
Supplement: Supplementary file 3 — Description of Additional Supplementary Files [file 41467_2020_14715_MOESM3_ESM.pdf]

Description of Additional Supplementary Files

File Name: Supplementary Movie 1

Description: The movie of the conventional fabrication process in ambient air at relative humidity of 41%. Transparent film was obtained after conventional dripping method, and rough perovskite film was obtained after annealing at 98 °C.

File Name: Supplementary Movie 2

Description: The movie of the prenucleation fabrication process in ambient air at relative humidity of 41%. Transparent film was obtained after first dripping, and brown film was obtained after second dripping. The brown film became smooth and black film after annealing at 98 °C.

File Name: Supplementary Movie 3

Description: The movie of the aging process of wet film fabricated by prenucleation method. The brown film obtained could slowly become smooth and black film in ambient air at room temperature without annealing.
